# Integrated Transcriptomic and Machine-Learning Analyses Identify Shared Na^+^ Overload-Related Gene Signatures in Inflammatory Bowel Disease and Ankylosing Spondylitis

**DOI:** 10.3390/genes17080938

**Published:** 2026-08-11

**Authors:** Luojin Wu, Chenghao Ou, Xuan Liu, Miaohan Yan, Jinghan Guan, Xinfeng Wang, Liming Mao, Qiuyun Xu, Zhaoxiu Liu

**Affiliations:** 1 Department of Immunology, Medical School of Nantong University, Nantong 226001, China; 2431310023@stmail.ntu.edu.cn (L.W.); och1964307273@outlook.com (C.O.); 2431310026@stmail.ntu.edu.cn (X.L.); 2531110370@stmail.ntu.edu.cn (M.Y.); 2531110377@stmail.ntu.edu.cn (J.G.); lmmao@ntu.edu.cn (L.M.); 2 Department of Gastroenterology and Hepatology, Affiliated Hospital of Nantong University, Medical School of Nantong University, Nantong 226001, China; 3 Department of Hematology, Affiliated Hospital of Nantong University, Medical School of Nantong University, Nantong 226019, China; wxf5204079@126.com; 4 Basic Medical Research Center, Medical School of Nantong University, Nantong 226019, China; 5 Jiangsu Province Key Laboratory in University for Inflammation and Molecular Drug Target, Nantong University, Nantong 226019, China

**Keywords:** necrosis by sodium overload, inflammatory bowel disease, ankylosing spondylitis, machine learning algorithm, drug-response prediction, molecular docking

## Abstract

**Background**: Ankylosing Spondylitis (AS) and inflammatory bowel disease (IBD) exhibit substantial pathophysiological overlap, including dysregulated innate immunity, barrier dysfunction, and Th17-mediated inflammation. However, whether Na^+^ overload-related genes (NRGs) exhibit shared transcriptional alterations in IBD and AS remains unclear. This study aimed to characterize the expression patterns of NRGs across IBD and AS and to identify candidate genes associated with both diseases. **Methods**: Differential expression analysis was performed to identify differentially expressed NRGs (DE-NRGs) in diseased tissues relative to normal tissues. Shared DE-NRGs between IBD and AS were screened and defined as common differentially expressed NRGs (Co-DE-NRGs). We then analyzed the correlations of these Co-DE-NRGs and explored their relationships with immune cell infiltration in target tissues. Four machine learning algorithms were applied to screen key NRGs associated with both IBD and AS. Potential therapeutic agents targeting these core biomarkers were predicted using drug–gene interaction databases, and molecular docking was conducted for further validation. **Results**: A total of 32 shared Co-DE-NRGs were identified for IBD and AS, with nine key regulatory NRGs recognized: *CALR*, *CD63*, *CYBA*, *DYSF*, *HYOU1*, *IL1B*, *JAK1*, *MMP9*, and *STAT3*. Exploratory MR analysis identified disease-specific associations between genetically predicted expression of NRGs and CD, UC, and AS. Genetically predicted *STAT3* expression showed positive associations with CD and UC but an inverse association with AS and therefore did not represent a consistent risk factor across the three diseases. Furthermore, transcriptome-based drug-response analysis identified four candidate agents shared between AS and at least one IBD dataset: ciclosporin, BCL-LZH-4, BRD-K79669418, and CID-5951923. Exploratory molecular docking generated STAT3 binding poses for BCL-LZH-4 and CID-5951923, with DOCK Grid Scores of −35.321896 and −28.361128, respectively. CID-5951923 was selected for representative visualization of its predicted interaction with STAT3. Single-cell RNA-sequencing analysis identified tissue- and cell-type-specific *STAT3* mRNA expression patterns in the analyzed IBD colonic and AS peripheral-blood datasets, with monocytes representing a major cell population exhibiting detected *STAT3* expression in the AS dataset. **Conclusions**: These findings identify shared NRG-related transcriptional alterations in IBD and AS, with *STAT3* emerging as a candidate gene associated with both diseases. Further experimental studies are required to determine whether these alterations reflect the involvement of NECSO and to evaluate their potential diagnostic or therapeutic relevance.

## 1. Introduction

Inflammatory bowel disease (IBD) is a chronic disorder characterized by persistent gastrointestinal inflammation, mainly consisting of Crohn’s disease (CD) and ulcerative colitis (UC) [1]. As a lifelong inflammatory illness, the incidence of CD has stabilized in many early-industrialized regions but continues to increase in newly industrialized regions, particularly in Asia [2,3]. The pathogenesis of both CD and UC is multifactorial, involving genetic susceptibility, environmental exposures, intestinal microbiota dysbiosis, and dysregulated innate and adaptive immune responses [4,5]. Currently, around 5 million people worldwide suffer from UC, and its incidence and hospitalization rates are growing rapidly in China, India and Latin America. Typical clinical symptoms of UC include hematochezia, tenesmus and abdominal pain. Although physical examinations show no obvious abnormalities in some patients, these symptoms greatly reduce patients’ quality of life [6,7]. The overall prevalence of IBD continues to rise worldwide. Most IBD patients still face delayed diagnosis and limited targeted treatment options. Therefore, exploring reliable predictive biomarkers for treatment response is urgently needed clinically, which can cut medical costs and reduce the social and personal disease burden caused by IBD [8].

Spondyloarthritis (SpA) encompasses a heterogeneous group of immune-mediated inflammatory diseases. Axial spondyloarthritis (axSpA) predominantly affects the sacroiliac joints and spine and includes radiographic axSpA, traditionally termed ankylosing spondylitis (AS), and non-radiographic axSpA, in which definite sacroiliitis is not visible on conventional radiographs. Clinically, axSpA commonly presents with inflammatory back pain, morning stiffness, and reduced spinal mobility; peripheral arthritis and enthesitis may also occur, together with extra-musculoskeletal manifestations such as acute anterior uveitis, psoriasis, and IBD. Persistent inflammation involving the sacroiliac joints, vertebral corners, and entheses may lead to erosive changes, syndesmophyte formation, and, in advanced disease, spinal ankylosis. Management combines regular exercise and physical therapy with pharmacological treatment. Non-steroidal anti-inflammatory drugs are generally used as initial therapy, whereas TNF inhibitors or IL-17 inhibitors—and, in selected cases, JAK inhibitors—are considered for persistently active disease. When IBD coexists, treatment selection should account for intestinal disease activity and should be coordinated with a gastroenterologist [9,10]. AS and IBD often occur together clinically. Epidemiological data show that 6–14% of AS patients develop secondary IBD, while 3.7–4.5% of IBD patients develop AS; in addition, 5–10% of AS patients are complicated with CD or UC [11,12]. Furthermore, this comorbidity rate is markedly higher than that observed in the general population. Patients with AS carry lifelong risk of IBD, and this risk increases with longer disease duration. Both diseases are chronic recurrent inflammatory disorders that affect joints and intestines respectively, and they share highly overlapping pathogenic mechanisms [13,14,15].

While IBD and AS are closely associated clinically, their specific pathophysiological mechanisms are yet to be clarified. Current treatments focus on individual diseases, and effective targets for comorbid patients are absent. Accumulating evidence indicates that IBD and AS share core immunological mechanisms. As typical immune-mediated inflammatory diseases (IMIDs), they share multiple common immune pathways, including the IL-23/Th17 axis, NF-κB, and JAK1/STAT3 signaling pathways [16,17,18,19]. Regulated cell death (RCD) can influence both the propagation and resolution of inflammation. RCD encompasses multiple mechanistically distinct modalities, including apoptosis, necroptosis, ferroptosis, pyroptosis, parthanatos, autophagy-dependent cell death, and lysosome-dependent cell death, among others. In particular, apoptosis facilitates the orderly elimination of activated or damaged cells. Prompt phagocytic clearance of apoptotic cells through efferocytosis limits secondary necrosis and the extracellular release of damage-associated molecular patterns, while promoting pro-resolving macrophage responses; conversely, defective clearance may perpetuate tissue inflammation. Among these RCD modalities, apoptosis, ferroptosis, and necroptosis have been investigated in relation to IBD and AS [20,21,22,23,24,25,26]. However, whether other poorly explored RCD modalities contribute to inflammatory transmission across the gut–joint axis remains to be determined.

**Figure 1:**
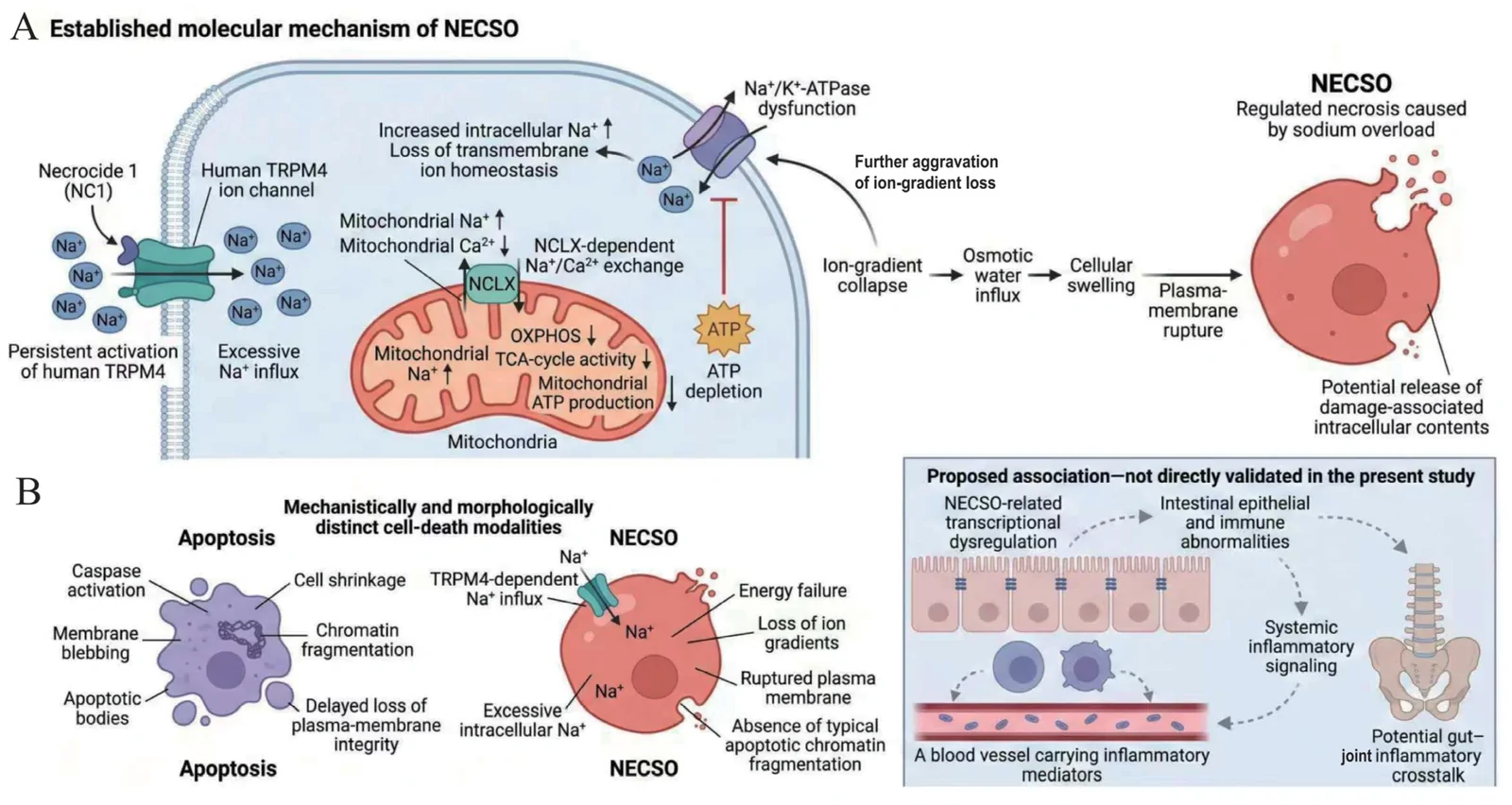
Molecular mechanism of necrosis by sodium overload and its potential relevance to the IBD-AS gut-joint axis. (**A**) Persistent activation of human transient receptor potential melastatin 4 (TRPM4) by necrocide 1 (NC1) drives excessive Na^+^ influx. The resulting increase in intracellular Na^+^ enhances NCLX-mediated mitochondrial Na^+^/Ca^2+^ exchange, leading to mitochondrial Na^+^ accumulation, mitochondrial Ca^2+^ depletion, impaired oxidative phosphorylation and tricarboxylic acid-cycle activity, and reduced ATP production. ATP depletion compromises Na^+^/K^+^-ATPase activity, further destabilizing transmembrane ion gradients and ultimately causing osmotic water influx, cellular swelling, plasma-membrane rupture, and NECSO. Membrane rupture may result in the release of damage-associated intracellular contents. (**B**) Morphological and mechanistic distinction between apoptosis and NECSO. Apoptosis is characterized by caspase activation, cell shrinkage, membrane blebbing, chromatin fragmentation, and the formation of apoptotic bodies, with plasma-membrane integrity maintained until the late stages. In contrast, NECSO is characterized by TRPM4-dependent Na^+^ influx, energy failure, loss of ion gradients, cellular swelling, and plasma-membrane rupture, without typical apoptotic chromatin fragmentation. The boxed inset presents a proposed model linking NECSO-related transcriptional dysregulation to intestinal epithelial and immune abnormalities, systemic inflammatory signaling, and potential gut-joint inflammatory crosstalk. Dashed arrows denote hypothesized associations that were not directly validated in the present study. Abbreviations: AS, ankylosing spondylitis; IBD, inflammatory bowel disease; NC1, necrocide 1; NCLX, mitochondrial Na^+^/Ca^2+^ exchanger; NECSO, necrosis by sodium overload; OXPHOS, oxidative phosphorylation; TCA, tricarboxylic acid; TRPM4, transient receptor potential melastatin 4.

Intracellular sodium overload has been implicated in fetal developmental abnormalities, renal dysfunction, cardiac arrhythmias, ischemia, and hyperosmotic stress [27,28,29,30]. Na^+^ by sodium overload (NECSO) is a recently described form of regulated necrosis. In the experimental model reported by Fu et al., persistent activation of human TRPM4 by necrocide 1 drives Na^+^ influx, loss of ion homeostasis, cellular swelling, and eventual plasma-membrane rupture [30]. These necrotic features distinguish NECSO from apoptosis, which typically involves caspase activation, cell shrinkage, chromatin fragmentation, and preservation of plasma-membrane integrity until the late stages. NECSO is also mechanistically distinct from necroptosis and ferroptosis because it does not require RIPK1/RIPK3/MLKL signaling or iron-dependent lipid peroxidation, respectively [30,31]. TRPM4 is a calcium-activated nonselective cation channel that is indispensable for sodium-dependent cell death progression [31]. Sustained TRPM4 activation induced by the chemical agonist Necrocide 1 (NC1) drives robust sodium influx, markedly elevates intracellular osmotic pressure, and ultimately triggers cell membrane rupture and NECSO occurrence [32,33]. Existing evidence has demonstrated that TRPM4 maintains intestinal ion homeostasis and modulates epithelial fluid balance through a Ca2^+^-independent activation manner, and dysfunction of this channel is tightly associated with intestinal mucosal barrier damage during colitis [34]. Although previous reviews have outlined the critical regulatory roles of TRP channels in IBD pathogenesis [35], the specific functions of TRPM4 and TRPM4-mediated NECSO in IBD remain poorly elucidated. The established molecular events underlying NECSO and its proposed relevance to the IBD–AS gut–joint axis are summarized in Figure 1.

Although chemical screening has confirmed that specific necroptosis inhibitors are capable of blocking cell death triggered by NC1 stimulation or energy depletion, the precise pathogenic mechanism of AS remains largely unclear, and few studies have focused on the relationship between necroptosis and AS [30]. These observations prompted us to investigate whether Na^+^ overload-related genes (NRGs) exhibit shared transcriptional alterations in IBD and AS. Because the datasets analyzed in this study do not contain direct measurements of TRPM4 activity, intracellular Na^+^ accumulation, or other cellular features required to establish NECSO, our analysis was designed to identify shared NRG-related molecular signatures rather than to establish NECSO as a mechanism linking the two diseases.

Based on the above research findings and mechanistic hypothesis, this study aims to address critical unresolved issues regarding the comorbidity of IBD and AS. Although emerging evidence implicates sodium overload in immune dysregulation, the current literature lacks a systematic characterization of how NRGs regulate the concurrent development of IBD and AS, thereby hindering a mechanistic understanding of their shared molecular association. To fill this research gap, we analyzed public transcriptomic datasets from intestinal tissues of CD [36,37] and UC [38,39] patients, as well as peripheral blood samples of AS patients [40,41]. First, we identified common differentially expressed NRGs (Co-DE-NRGs) exhibiting concordant dysregulation across both IBD subtypes and AS. Subsequently, we integrated immune-infiltration analysis, machine learning, exploratory MR, single-cell transcriptomics, and drug-response prediction to prioritize cross-disease candidate genes. This multi-layer framework represents a distinguishing feature of our study and provides a hypothesis-generating basis for investigating NRG-related molecular alterations across the IBD–AS gut–joint axis and selecting candidates for subsequent experimental validation. It proposes shared molecular features of the two diseases and provides possible biomarkers and therapeutic targets for further basic research and clinical treatment.

## 2. Materials and Methods

### 2.1. Data Source

Transcriptomic datasets in this study were obtained from the Gene Expression Omnibus (GEO) database. CD and UC samples were from human colon biopsies. CD analysis used GSE16879 (6 healthy controls, 37 CD patients) [36], GSE36807 (7 healthy controls, 13 CD patients) [38], GSE179285 (23 healthy controls, 86 CD patients) [38], and GSE59071 (11 healthy controls, 8 CD patients) [28]. UC analysis included GSE13367 (20 healthy controls, 34 UC patients) [29], GSE59071 (11 healthy controls, 97 UC patients) [38], GSE75214 (11 healthy controls, 97 UC patients), and GSE179285 (23 healthy controls, 55 UC patients) [38]. AS research used GSE18781 (25 healthy controls, 18 AS patients) [40] and GSE25101 (16 healthy controls, 16 AS patients) [41]. CD, UC, and AS datasets were not pooled into a single expression matrix. Instead, integration was performed separately for the CD colonic, UC colonic, and AS peripheral-blood datasets. Each GEO dataset was normalized separately before integration, and GEO accession was specified as the batch variable in the ComBat algorithm implemented in the sva R package (version 3.52.0) [42]. Principal component analysis was performed before and after adjustment to evaluate accession-associated variation (Supplementary Figure S1). ComBat was used to reduce major technical differences among datasets rather than to assume the complete elimination of cross-study heterogeneity.

### 2.2. Identification of DE-NRGs

Na^+^ overload-related genes (NRGs) were retrieved from the GeneCards database (https://www.genecards.org/; accessed on 23 April 2026) using the exact search term “Na^+^ overload-induced necrotic death.” The search returned 263 unique genes. No additional relevance-score cutoff was applied, and all returned genes were retained as the initial NRG set. The GeneCards relevance scores ranged from 1.0068 to 62.2399. The complete list of NRGs, including gene symbols, descriptions, categories, and GeneCards relevance scores, is provided in Supplementary Table S1.

Before differential expression analysis, the 263-gene list was matched to the genes represented in each normalized expression matrix, and only NRGs with available expression measurements were included in the corresponding dataset-specific analysis. Comparisons between disease and control samples were performed separately for the CD, UC, and AS datasets using the Wilcoxon rank-sum test. To account for multiple testing, *p* values were adjusted within each dataset across all NRGs with valid expression measurements using the Benjamini–Hochberg procedure. A Benjamini–Hochberg-adjusted *p* value < 0.05 was considered statistically significant. Both nominal and adjusted *p* values for all tested NRGs are provided in Supplementary Table S2. For exploratory cross-disease screening, genes meeting the nominal criteria of *p* < 0.05 and |log2FC| > 1 were retained and compared across datasets. Genes identified using these nominal criteria were considered exploratory candidates rather than FDR-confirmed differentially expressed genes. Gene-expression patterns were visualized as heatmaps using the pheatmap R package (v1.0.13) [43]. DE-NRGs identified in both the IBD and AS analyses were subsequently defined as common differentially expressed NRGs (Co-DE-NRGs). Pairwise Pearson correlation analysis was performed to characterize the co-expression relationships among these Co-DE-NRGs, and the resulting correlation matrices were visualized using the corrplot package (v0.95) [44].

### 2.3. Immune Cell Infiltrations Analysis

Immune-cell composition was estimated from the normalized bulk gene-expression matrices using CIBERSORT implemented in IOBR (v0.99.9). CIBERSORT applies nu-support vector regression to deconvolve each bulk expression profile against the LM22 reference matrix, which contains multigene signatures for 22 human leukocyte phenotypes. T-lymphocyte subsets and regulatory T cells were inferred from their composite LM22 expression signatures rather than from individual markers such as CD3 or FOXP3. The estimated relative fractions of the 22 immune-cell subsets were compared between control and disease samples using nonparametric tests. Pearson correlation analysis was subsequently performed between the expression levels of the 32 Co-DE-NRGs and the estimated immune-cell fractions within each disease-specific dataset. Correlation results were visualized using ggplot2 (v3.5.1) [45], aiding in the identification of immune subsets impacted by dysregulated sodium-overload-related transcription.

### 2.4. Machine Learning-Based Screening of Candidate Genes

Machine-learning techniques were utilized to screen for potential biomarkers and identify Co-DE-NRGs with strong discriminatory power across different disease states. Four diverse algorithms—Support Vector Machine (SVM) [46], Random Forest (RF) [47].

### 2.5. Differential Analysis and Gene Set Variation Analysis (GSVA) Between Subgroups

Generalized Linear Model (GLM) [48], and K-Nearest Neighbors (KNN) [49]—were applied separately to the CD, UC, and AS datasets. Gene-expression features were standardized using Z-score normalization, after which the samples were randomly divided into training and test sets at a ratio of 75:25. Five-fold cross-validation was conducted within the training set, and genes were ranked according to the feature-selection results of each algorithm. The 15 highest-ranked genes from each algorithm were compared, and genes retained across multiple algorithms were considered exploratory candidate genes. This initial analysis was used for candidate-gene prioritization rather than for external validation.

To provide a more stringent assessment of cross-cohort performance, the analysis was subsequently expanded to 113 pipelines constructed from 12 algorithms: Lasso, Ridge, elastic net, stepwise generalized linear modeling, SVM, glmBoost, linear discriminant analysis, partial least-squares generalized linear modeling, RF, gradient boosting machine, XGBoost, and naive Bayes. Distinct GEO accessions were assigned to model development and held-out evaluation. For CD, GSE16879, GSE36807, and GSE59071 were used for model development, whereas GSE179285 was held out for evaluation. For UC, GSE13367, GSE59071, and GSE75214 were used for model development, whereas GSE179285 was held out. For AS, GSE18781 was used for model development, whereas GSE25101 was held out. Each GEO dataset was normalized separately before integration. ComBat harmonization was then performed within each disease-specific expression matrix to reduce study- and platform-related batch variation. After the model-development and held-out matrices had been generated, Z-score standardization was performed separately within the two sets.

Candidate models were fitted using the model-development cohorts and evaluated in the corresponding held-out GEO cohorts. Pipelines retaining no more than five features were excluded, resulting in 102, 113, and 59 evaluable pipelines for CD, UC, and AS, respectively. Model performance was assessed using the area under the receiver operating characteristic curve with 95% confidence intervals, together with accuracy, sensitivity, specificity, precision, and F1 score. For exploratory comparison, the displayed models were selected according to their overall performance across the model-development and held-out cohorts. Because the held-out-cohort performance contributed to this comparison, and because the held-out samples contributed to the preceding unsupervised ComBat harmonization, the analysis was defined as GEO cohort-held-out evaluation rather than fully independent external validation.

The samples were divided into *STAT3^+^* and *STAT3^−^* groups based on their median *STAT3* values. Differential gene expression analysis was performed using limma-voom to identify genes that were differentially expressed between the two groups. Volcano plots were generated using ggplot2 (v3.5.1). Subsequently, GSVA (v1.36.2) was utilized to calculate pathway activity scores for each sample using BP and KEGG gene sets from MSigDB. A pathway score matrix was constructed for further analysis. Limma was then employed to compare GSVA scores between the *STAT3^+^* and *STAT3^−^* groups, with pathways showing |logFC| > 2 and adjusted *p* < 0.05 considered significantly enriched.

### 2.6. Drug Prediction

Transcriptome-based drug-response prediction was performed separately for the CD, UC, and AS cohorts using the oncoPredict R package (v0.2). Gene-expression profiles and drug-response data from the Cancer Therapeutics Response Portal version 2 (CTRP2) were used as the training reference. Drug-response scores were predicted using the calcPhenotype function with empirical Bayes batch correction, phenotype power transformation, removal of the lowest 20% of low-variance genes, and a minimum sample size of 10. Pearson correlation coefficients were calculated between the expression levels of disease-associated differentially expressed NRGs and the predicted drug-response scores. *p* values were adjusted using the Benjamini–Hochberg method. Candidate agents were prioritized according to the direction and coverage of their correlations with the disease-associated genes. The coverage thresholds used in the current analysis were 90% for CD, 80% for UC, and 60% for AS. Agents shared between AS and either CD or UC were retained for subsequent evaluation.

### 2.7. Molecular Docking Analysis

The crystal structure of human STAT3 was obtained from the RCSB Protein Data Bank (PDB ID: 6TLC). Chain A, corresponding to STAT3 residues 136-689, was retained for docking. Candidate ligand structures were obtained from PubChem. Receptor and ligand preprocessing, including removal of non-receptor components and preparation of the docking structures, was performed using UCSF Chimera (v1.19). Molecular docking was conducted using UCSF DOCK 6.11 with the flexible anchor-and-grow algorithm. The docking region was defined using 48 selected spheres, with a grid spacing of 0.3 Å and a grid-box center of −21.552, 22.458, and −20.835 Å. The corresponding grid-box dimensions were 33.404 × 37.111 × 33.113 Å. A maximum of 1000 ligand orientations was evaluated. The DOCK Grid Score was used to rank the predicted poses. The resulting complexes were inspected and visualized using PyMOL (v3.0.5).

### 2.8. scRNA-Seq Analysis

In this study, we selected the single-cell dataset GSE214695 (comprising 6 control samples, 6 CD colon samples, and 6 UC colon samples), GSE232131 (comprises six peripheral blood mononuclear cell samples from patients with ankylosing spondylitis, including three unstimulated samples and three ex vivo immune-stimulated samples.) for analysis. A total of 46,700 cells passed initial quality control and were processed using the Seurat R package (v5.3.0). All data from the collected samples were combined into a single Seurat object. Following this integration, cells of low quality were excluded according to established thresholds for both mitochondrial gene content and the total number of genes detected per cell. We then performed principal component analysis (PCA) to evaluate major sources of variation and determine the optimal number of principal components for downstream analyses. UMAP was subsequently used for dimensionality reduction and visualization. Major cell types were annotated using the SingleR package (v2.4.1). Because the datasets were derived from different tissue compartments, their cellular and expression patterns were summarized within the corresponding dataset and were not interpreted as directly comparable across diseases. Within each cell type, cells were categorized as “*STAT3*-detected” when normalized *STAT3* expression was >0 and “*STAT3*-undetected” when normalized expression was equal to 0. The FindMarkers function was employed to identify DEGs, with significance thresholds set at *p* < 0.05 and |log_2_ fold-change| > 1. Because zero values in sparse scRNA-seq data may reflect either low expression or transcript dropout, this classification was used to characterize transcript-detection patterns and was not interpreted as a measure of *STAT3* activation or biological positivity.

### 2.9. Mendelian Randomization Analysis

We used the TwoSampleMR R package (v0.6.18) to conduct two-sample Mendelian randomization (MR) analyses examining the associations between genetically predicted expression of Co-DE-NRGs and the risks of CD, UC, and AS. Summary-level whole-blood eQTL data for the Co-DE-NRGs were obtained from the eQTLGen resource through the IEU OpenGWAS database (eqtl-a-ENSG…) and were used as the exposures. This resource contains cis-eQTLs and a subset of trans-eQTLs associated with gene-expression levels in whole blood. For CD, five genome-wide association studies (GWAS) were included (ebi-a-10, ieu-a-12, ieu-a-30, ebi-a-GCST003044, finn-b-K11_CROHN), and for UC, eight GWAS datasets were used (ebi-a-32, ieu-a-970, ieu-a-973, ieu-a-968, finn-b-K11_ULCER, ebi-a-GCST90018933, ebi-a-GCST003045, ebi-a-GCST000964). In addition, five AS GWAS datasets (ebi-a-GCST005529, finn-b-M13_ANKYLOSPON, finn-b-M13_ANKYLOSPON_STRICT, ukb-a-88, ukb-b-18194) were retrieved from the IEU OpenGWAS database (https://gwas.mrcieu.ac.uk/) (accessed on 15 May 2026). Application of the conventional genome-wide significance threshold (*p* < 5 × 10^−8^) left too few independent variants for many gene-outcome pairs to support multi-instrument analyses. Therefore, SNPs associated with the expression of each Co-DE-NRG at *p* < 5 × 10^−4^ were selected as IVs for this exploratory analysis. To ensure independence, SNPs were clumped using an LD threshold of r^2^ < 0.001 within a 10,000 kb window. Instrument strength was assessed using F-statistic, and SNPs with F < 10 were excluded to minimize weak-instrument bias. Exposure and outcome alleles were harmonized using harmonise_data (…, action = 2). Allele-frequency information was used to resolve palindromic SNPs where possible, whereas ambiguous variants were excluded. Gene-outcome pairs with fewer than two harmonized IVs were not included in the multi-instrument MR analysis. The inverse-variance weighted (IVW) method was used as the primary estimator of the genetically predicted gene expression-disease associations. MR-Egger regression, the weighted-median method, simple mode, and weighted mode were additionally applied as sensitivity estimators. Horizontal pleiotropy was evaluated using MR-Egger intercept, and heterogeneity among instruments was assessed via Cochran’s Q statistic. MR-PRESSO was used to assess global pleiotropy and identify potential outliers. Leave-one-out analyses were performed to examine whether any single SNP disproportionately influenced the MR estimates. Given the relaxed instrument-selection threshold and the inclusion of both cis- and trans-eQTLs, the MR findings were considered exploratory and hypothesis-generating.

### 2.10. Statistical Analysis

All statistical analyses were performed using R software (v4.4.2). For comparisons between two groups, the Wilcoxon rank-sum test was applied. Correlation analyses were conducted using Pearson correlation coefficients. Unless otherwise specified, statistical significance was defined as *p* < 0.05 and |log_2_FC| = >1.

## 3. Results

### 3.1. Identification of Co-DE-NRGs in IBD and AS

To determine whether NRGs exhibit shared transcriptional alterations in IBD and AS, we systematically identified and characterized differentially expressed NRGs across the analyzed datasets. This cross-disease analysis was intended to identify conserved expression patterns associated with the two diseases and to generate hypotheses for subsequent functional investigation.

We retrieved eight independent, peer-reviewed gene expression datasets from the GEO database. Four intestinal tissue datasets containing healthy controls and CD samples, namely GSE16879, GSE36807, GSE179285 and GSE59071, were merged to establish a combined control-CD dataset. Similarly, GSE13367, GSE59071, GSE75214 and GSE179285 were integrated to build a unified control-UC dataset. For AS, we combined GSE18781 and GSE25101 (including healthy controls and AS peripheral blood samples) to generate a consolidated control-AS dataset (Supplementary Figure S1).

**Figure 2:**
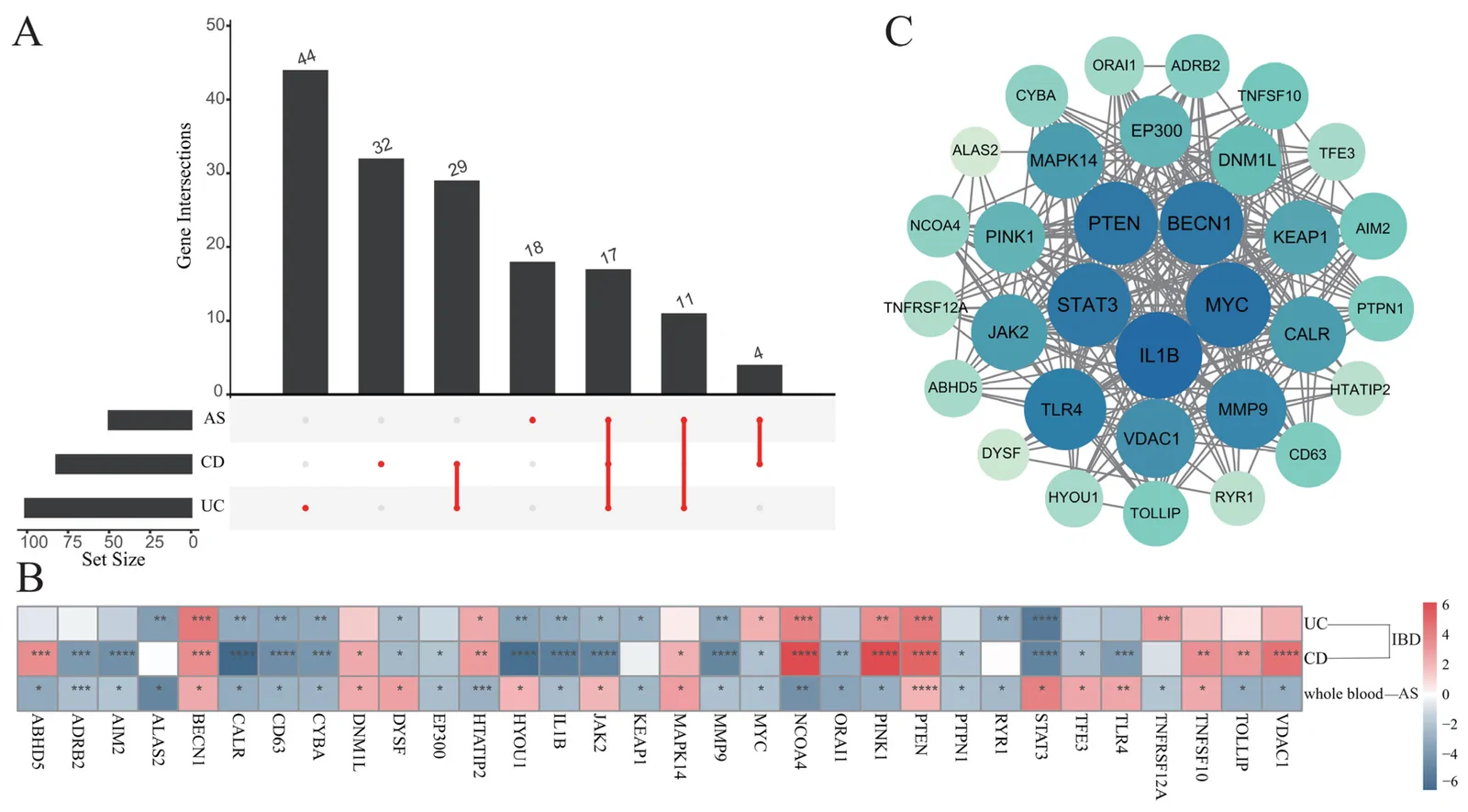
Identification of Co-DE-NRGs associated with CD, UC, and AS. (**A**) Upset plot illustrating the distribution of DEGs in CD, UC, and AS. (**B**) Heatmap of the expression profiles of 32 Co-DE-NRGs in CD, UC, and whole blood samples. (**C**) The protein–protein interaction network of 32 Co-DE-NRGs. *: *p* < 0.05; **: *p* < 0.01; ***: *p* < 0.001; ****: *p* < 0.0001.

The GeneCards search identified 263 unique NRGs, which were included as the initial candidate gene set (Supplementary Table S1). After matching these genes to the normalized expression matrices and performing differential expression analysis, we identified 32 Co-DE-NRGs exhibiting shared differential-expression patterns across the IBD and AS datasets. Their distribution and expression profiles are shown in the UpSet plot and heatmap, respectively (Figure 2A and Figure 2B). To further investigate the functional interactions among these overlapping genes, we developed a protein–protein interaction (PPI) network using the STRING database. Network analysis identified five core hub proteins: PTEN, BECN1, STAT3, MYC, and IL1B (Figure 2C).

### 3.2. Correlation Analysis of the 32 Co-DE-NRGs

**Figure 3:**
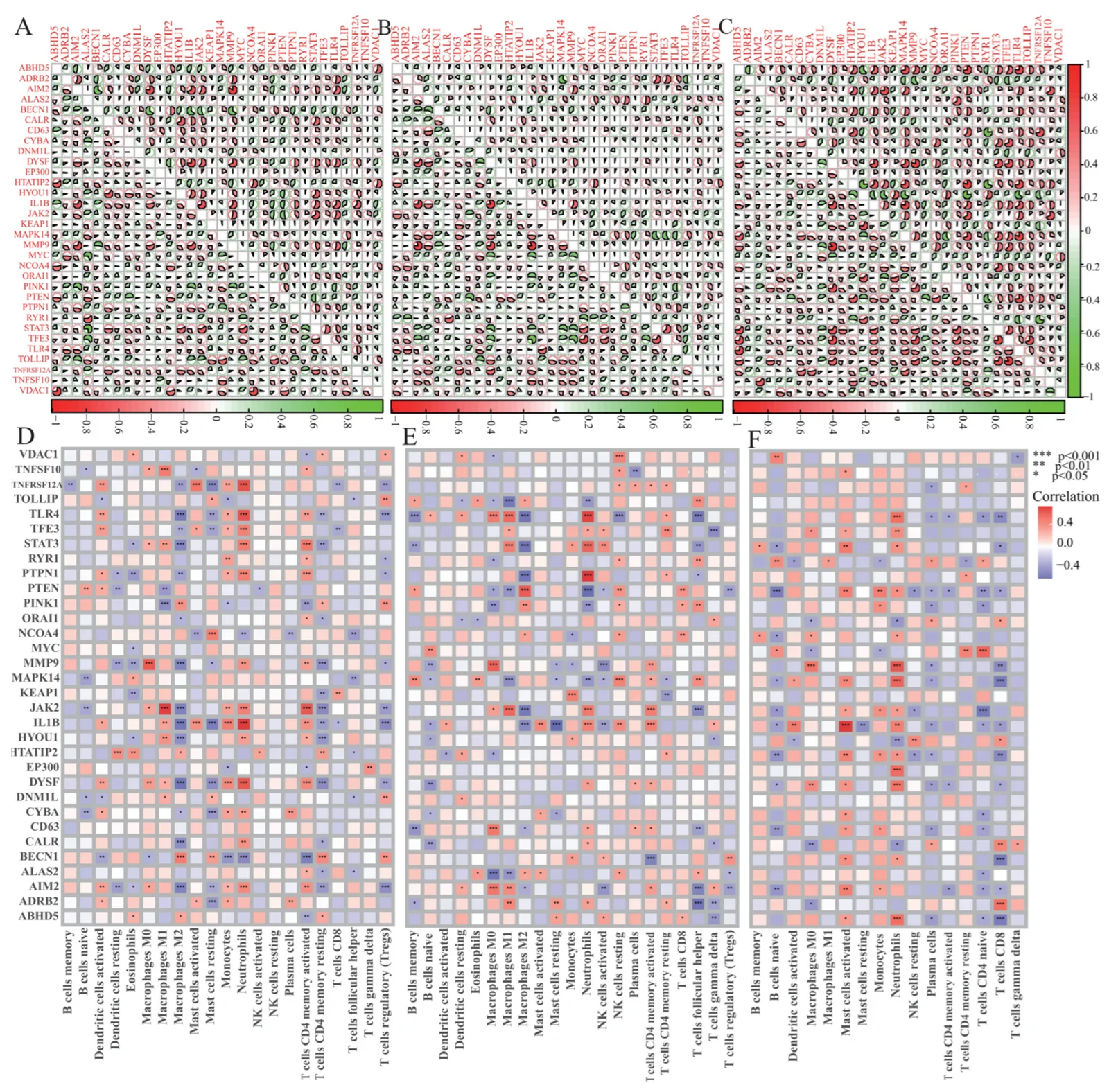
Correlations between the 32 Co-DE-NRGs and immune cell infiltration across three sample types: (**A**) CD colon, (**B**) UC colon, (**C**) AS whole blood. The correlations between 32 Co-DE-NRGs and immune cell infiltration (**D**) CD colon, (**E**) UC colon, and (**F**) AS whole blood.

To characterize disease-associated changes in co-expression, we calculated pairwise correlations among the 32 Co-DE-NRGs separately in healthy control and disease samples for CD, UC, and AS. This analysis aimed to clarify the regulatory interactions of the 32 Co-DEGs. The pairwise correlation matrices for the control and disease groups are presented in Figure 3A–C. In these correlation plots, healthy control samples predominantly exhibited negative correlations, indicated by green shading in the lower-left quadrant, whereas patient samples displayed primarily positive correlations, marked by red shading in the upper-right quadrant.

The 32 Co-DE-NRGs displayed pronounced tissue- and disease-specific heterogeneity in their co-expression patterns. The onset of disease significantly altered the linear correlations among these Co-DE-NRGs. In comparison to healthy controls, samples from individuals with IBD exhibited markedly strengthened negative correlations among several hub genes, including *CALR*, *CD63*, *CYBA*, *DYSF*, *HYOU1*, *IL1B*, *JAK1*, *MMP9*, and *STAT3* (Figure 3A,B). This observation indicates a disease-specific reconfiguration of gene regulatory interactions. Collectively, the co-expression patterns of the 32 shared DEGs vary considerably between physiological and pathological states, highlighting extensive remodeling of their intrinsic regulatory networks in inflammatory diseases.

### 3.3. Correlation Analysis Between 32 Co-DE-NRGs and Immune Cells

To further investigate whether shared NRG-related transcriptional signatures are associated with immune dysregulation, we examined the correlations between the 32 Co-DE-NRGs and immune cell infiltration in colon tissues from patients with CD and UC, as well as in whole blood from individuals with AS (Figure 3D–F). In all three conditions, the core genes *TLR4*, *TFE3*, *STAT3*, *IL1B*, and *DYSF* exhibited positive correlations with neutrophils and negative correlations with resting mast cells. In the colon tissues of CD and UC, these Co-DE-NRGs demonstrated strong negative correlations with M2 macrophages and naive B cells. Conversely, in AS peripheral blood, Co-DE-NRGs were positively associated with myeloid inflammatory cells but negatively associated with CD8^+^ T cells and plasma cells. These findings suggest that Co-DE-NRGs may facilitate the progression of IBD and AS by altering immune cell recruitment and infiltration.

### 3.4. Machine Learning Screening for the Important Genes

#### 3.4.1. Initial Four-Algorithm Screening and Candidate-Gene Prioritization

**Figure 4:**
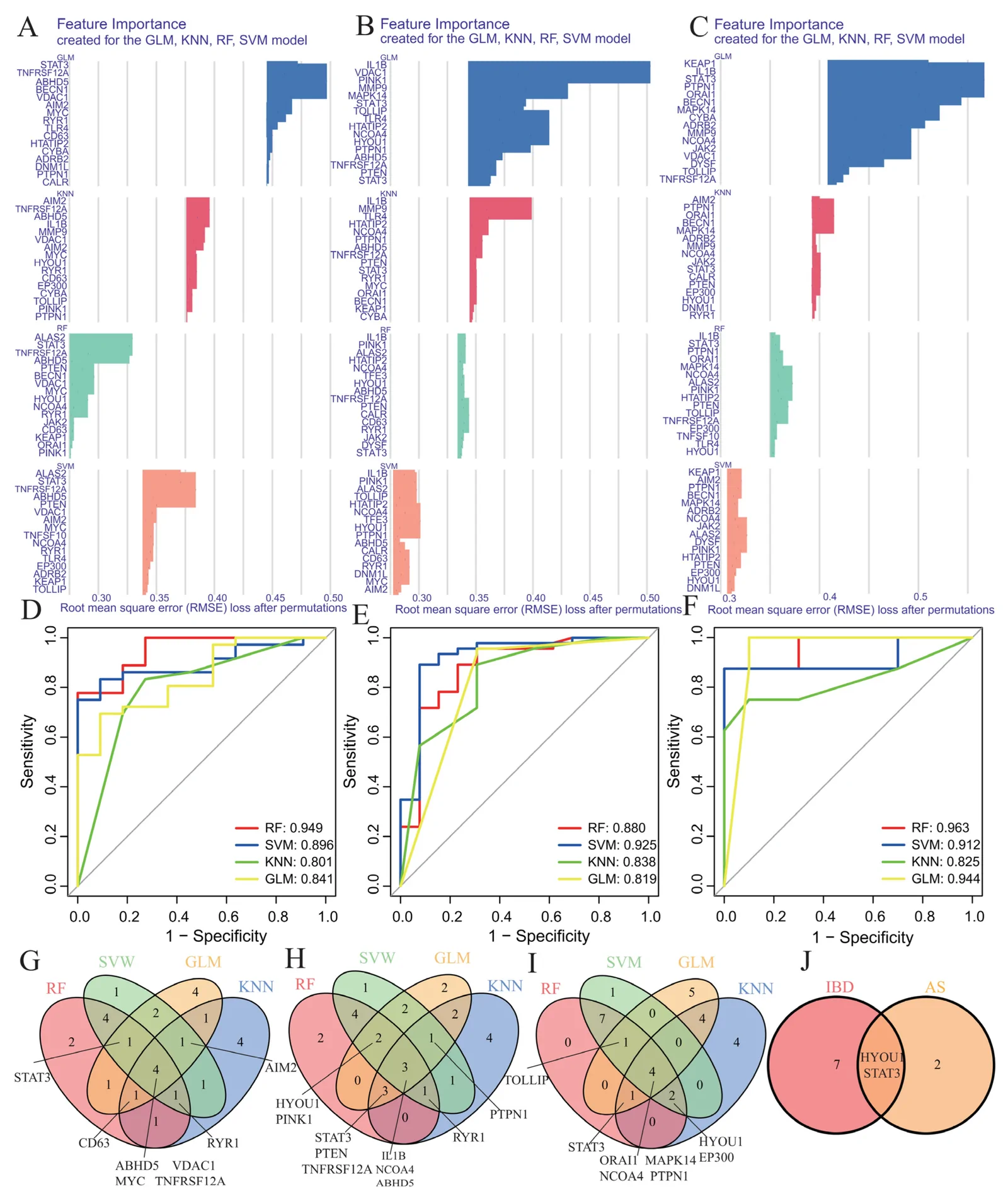
Machine-learning identification of key Co-DE-NRGs associated with CD, UC, and AS. The top 15 Co-DE-NRGs for (**A**) CD colon samples, (**B**) UC colon samples, and (**C**) AS whole blood samples were identified using GLM, RF, SVM, and KNN. (**D**–**F**) ROC curves of CD, UC and AS whole blood. Venn diagrams showing the overlapping key genes detected by the four algorithms in (**G**) CD colon, (**H**) UC colon, and (**I**) AS whole blood. (**J**) Venn diagram illustrating the shared genes across CD, UC, and AS whole blood.

To identify NRGs associated with disease status, we subsequently employed multiple machine-learning algorithms to prioritize Co-DE-NRGs with discriminatory value across the IBD and AS datasets. Our modeling efforts concentrated on colon tissues from patients with CD or UC, as well as whole-blood samples from individuals with AS. Utilizing support vector machine (SVM), random forest (RF), k-nearest neighbors (KNN), and generalized linear model (GLM) algorithms, we identified the 15 highest-ranked Co-DE-NRGs for CD (Figure 4A), UC (Figure 4B), and AS whole blood (Figure 4C).

In this initial discovery-stage analysis, we evaluated the apparent discriminatory performance of the four algorithms by constructing receiver operating characteristic (ROC) curves and calculating the area under the curve (AUC). For CD, the AUC values were 0.949, 0.896, 0.801, and 0.841 for RF, SVM, KNN, and GLM, respectively (Figure 4D). For UC, the corresponding AUC values were 0.880, 0.925, 0.838, and 0.819 (Figure 4E). For AS whole blood, the AUC values were 0.963, 0.912, 0.825, and 0.944, respectively (Figure 4F). The AUC results were consistent with the additional classification metrics, including accuracy, precision, recall, and F1 score. In the original random-split analysis, RF and SVM showed comparatively strong performance in CD, SVM showed the highest performance in UC, and RF showed the highest performance in AS. Because these estimates were obtained from the original sample-level random split, they were used only for preliminary comparison of the four algorithms and were not considered evidence of external model generalizability.

Venn diagrams were then used to identify candidate genes retained by three or more algorithms in CD (Figure 4G), UC (Figure 4H), and AS whole blood (Figure 4I). The candidate genes identified for CD included *STAT3*, *CD63*, *ABHD5*, *VDAC1*, *MYC*, *TNFRSF12A*, *RYR1*, and *AIM2*. The corresponding candidate genes for UC were *HYOU1*, *PINK1*, *STAT3*, *PTEN*, *TNFRSF12A*, *IL1B*, *NCOA4*, *ABHD5*, *RYR1*, and *PTPN1*, whereas those identified for AS were *TOLLIP*, *STAT3*, *ORAI1*, *MAPK14*, *NCOA4*, *PTPN1*, *HYOU1*, and *EP300*. *STAT3* was retained across the CD, UC, and AS analyses, whereas *HYOU1* was shared between the UC and AS analyses (Figure 4J). These genes were therefore considered exploratory cross-disease candidates rather than validated diagnostic biomarkers.

#### 3.4.2. Cohort-Held-Out Evaluation of Machine-Learning Pipelines

To provide a more stringent assessment of model performance, we subsequently expanded the analysis to 113 prespecified machine-learning pipelines and replaced the original sample-level random split with GEO accession-defined cohort-held-out evaluation. After excluding pipelines that retained no more than five features, 102, 113, and 59 valid pipelines were evaluated for CD, UC, and AS, respectively (Supplementary Figure S2). For exploratory model comparison, the pipelines were ranked according to their overall AUC performance across the model-development and held-out cohorts. XGBoost, random forest, and Stepglm[backward]+RF were selected as the representative models for CD, UC, and AS, respectively.

The CD XGBoost model achieved an AUC of 0.994 (95% CI: 0.980–1.000) in the model-development cohort and 0.813 (95% CI: 0.708–0.901) in the held-out GSE179285 cohort (Supplementary Figure S3A,B). The UC random-forest model yielded an AUC of 0.951 (95% CI: 0.924–0.973) in the model-development cohort and 0.832 (95% CI: 0.733–0.915) in the held-out GSE179285 cohort (Supplementary Figure S3F). For AS, the Stepglm[backward]+RF model achieved an AUC of 0.956 (95% CI: 0.891–1.000) in the model-development cohort and 0.773 (95% CI: 0.594–0.926) in the held-out GSE25101 cohort (Supplementary Figure S3I,J).

Confusion matrix analysis was subsequently performed to further characterize model performance (Supplementary Figure S3C,D,G,H,L). In the held-out cohorts, the CD, UC, and AS models achieved accuracies of 70.6%, 71.8%, and 68.8%, respectively. Their corresponding sensitivities were 69.8%, 70.9%, and 81.3%, whereas their specificities were 73.9%, 73.9%, and 56.3%, respectively. Precision and F1 score were 90.9% and 78.9% for CD, 86.7% and 78.0% for UC, and 65.0% and 72.2% for AS, respectively. Complete performance metrics for both the model-development and held-out cohorts are provided in Supplementary Table S3. Although all three representative models retained discriminatory ability in their respective held-out cohorts, their performance was lower than that observed in the model-development cohorts. These results provide a more conservative estimate of cross-cohort performance.

#### 3.4.3. Feature-Level Evaluation and Cross-Disease Candidate Prioritization

The genes retained by the representative models were subsequently examined in the corresponding model-development cohorts (Supplementary Figure S4). The displayed genes retained by the CD XGBoost model included *STAT3*, *PTEN*, *BAK1*, *CHKA*, *CYBA*, *JAK2*, *HIF1A*, *ADM*, *HYOU1*, and *IL1B* (Supplementary Figure S4A). Among these genes, *STAT3* showed the highest individual AUC of 0.894 (Supplementary Figure S4B). The displayed genes retained by the UC random-forest model included *CYCS*, *CHKA*, *ADM*, *ACTA2*, *ISG15*, *HIF1A*, *STAT3*, *IL1B*, *JAK1*, and *JAK2* (Supplementary Figure S4C). *CYCS* showed the highest individual AUC of 0.844, whereas *STAT3* achieved an AUC of 0.752 (Supplementary Figure S4D). For AS, the Stepglm [backward]+RF model retained *PTEN*, *HTATIP2*, *HYOU1*, *CYCS*, *CD63*, *STAT3*, *ATF3*, and *ALPP* (Supplementary Figure S4E). *PTEN* showed the highest individual AUC of 0.822, followed by *HTATIP2* with an AUC of 0.791, whereas *STAT3* showed more limited individual discrimination, with an AUC of 0.635 (Supplementary Figure S4F).

The retention of *STAT3* in all three representative models was consistent with its identification in the initial four-algorithm analysis. However, its individual discriminatory performance differed across diseases and was particularly limited in AS. *STAT3* was therefore prioritized as an exploratory cross-disease candidate for subsequent integrative analyses rather than as a universally validated diagnostic biomarker.

### 3.5. Results of the Mendelian Randomization Analysis

**Figure 5:**
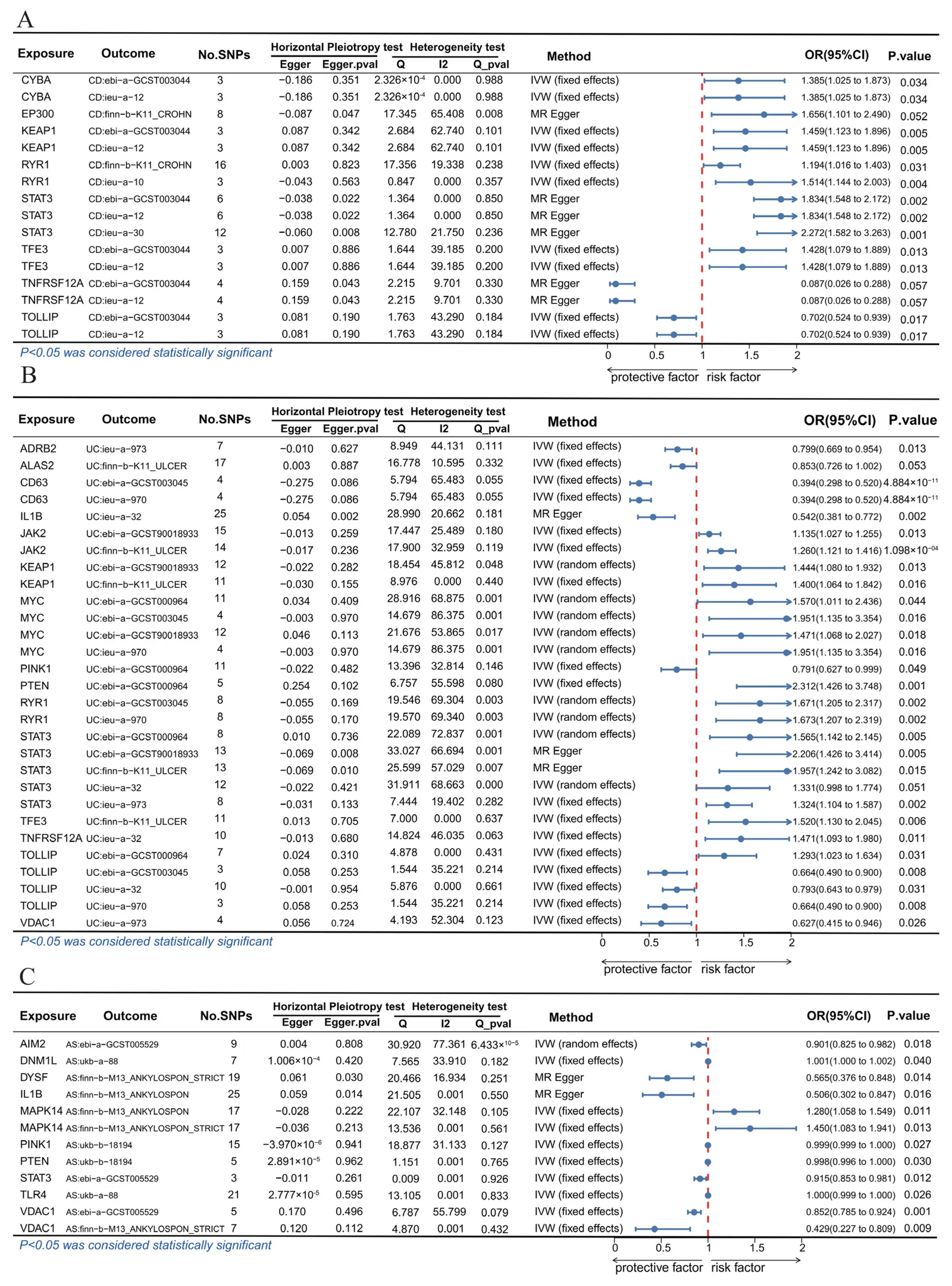
Mendelian randomization (MR) analysis evaluating the associations between genetically predicted expression of Co-DE-NRGs and the risks of (**A**) CD, (**B**) UC, and (**C**) AS.

To investigate associations between genetically predicted gene expression and these diseases, we performed Mendelian randomization (MR) analyses for CD (Figure 5A), UC (Figure 5B), and AS (Figure 5C) using the inverse-variance weighted (IVW) method. In CD, genetically predicted expression of *CYBA*, *EP300*, *KEAP1*, *RYR1*, *STAT3*, and *TFE3* showed positive associations with disease risk, whereas genetically predicted expression of *TNFRSF12A* and *TOLLIP* showed inverse associations (Figure 5A). For UC, positive associations were observed for *JAK2*, *KEAP1*, *MYC*, *PTEN*, *RYR1*, *STAT3*, *TFE3*, and *TNFRSF12A*, whereas *ADRB2*, *ALAS2*, *CD63*, *IL1B*, *PINK1*, *TOLLIP*, and *VDAC1* showed inverse associations (Figure 5B). In AS, genetically predicted *MAPK14* expression showed a positive association with disease risk, whereas *AIM2*, *DYSF*, *IL1B*, *STAT3*, and *VDAC1* showed inverse associations (Figure 5C). Collectively, these estimates revealed both shared and disease-specific patterns of association between genetically predicted expression of Co-DE-NRGs and the risks of CD, UC, and AS. Notably, genetically predicted *STAT3* expression showed positive associations with CD and UC but an inverse association with AS, highlighting a disease-specific pattern of genetic association. *IL1B* and *VDAC1* showed inverse associations with both UC and AS, further prioritizing them as cross-disease candidate genes for subsequent investigation. Overall, the exploratory MR analysis identified several Co-DE-NRGs with disease-specific association patterns and provided genetic evidence supporting further investigation of their potential roles in IBD and AS. These estimates should be interpreted within the instrumental-variable assumptions and validated in subsequent functional studies.

### 3.6. Comparison and Analysis of STAT3^+^ and STAT3^−^ Groups

**Figure 6:**
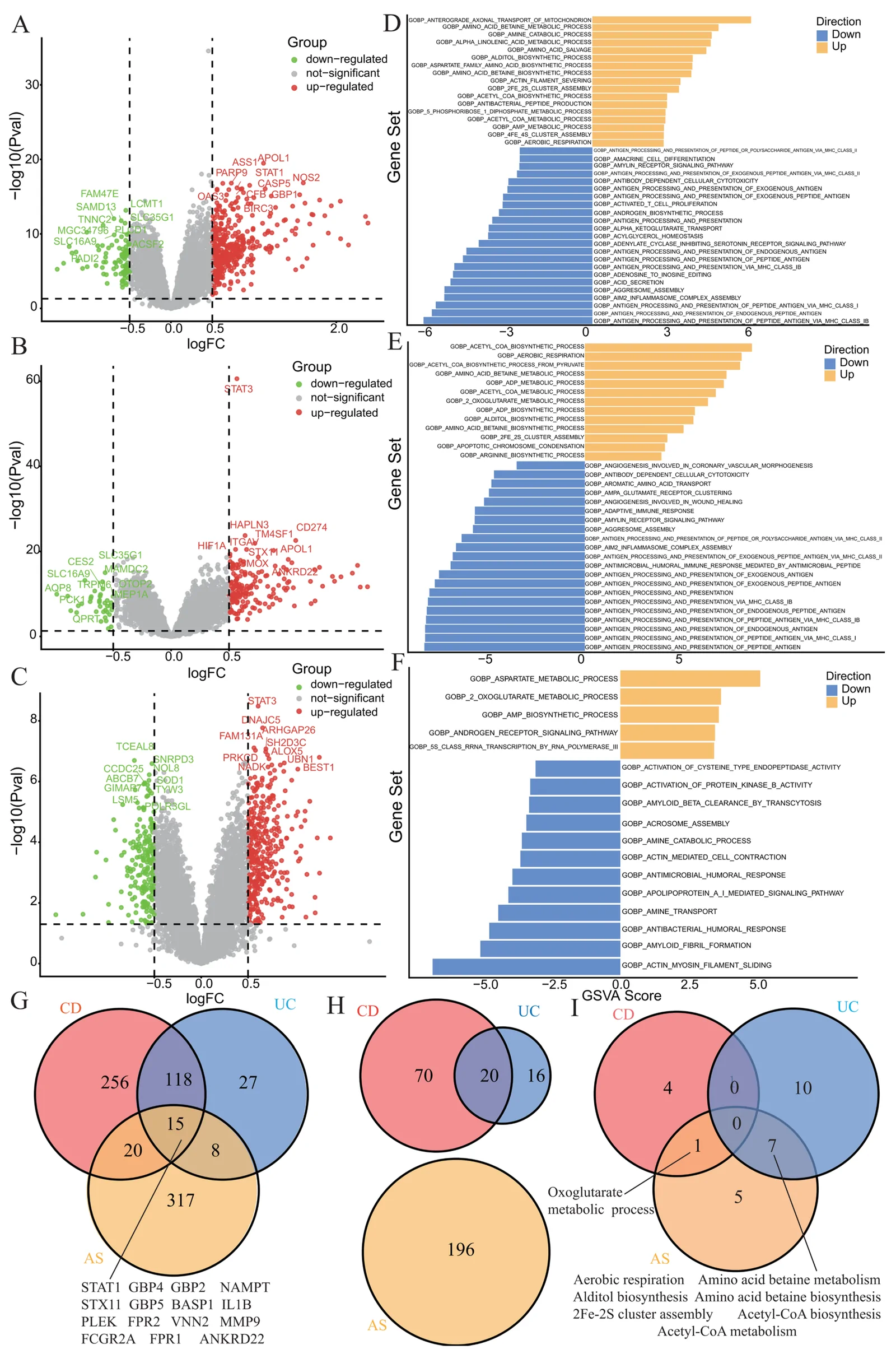
Differential expression and KEGG pathway analyses between *STAT3^+^* and *STAT3^−^* groups in CD, UC, and AS. (**A**–**C**) Volcano plots showing DEGs between *STAT3*^+^ and *STAT3*^−^ groups in (**A**) CD, (**B**) UC, and (**C**) AS whole blood. (**D**–**F**) GO and KEGG pathways differentially enriched between *STAT3^+^* and *STAT3^−^* groups in (**D**) CD, (**E**) UC, and (**F**) AS whole blood. (**G**–**I**) Venn diagrams illustrating the overlap of (**G**) upregulated and (**H**) downregulated DEGs among CD, UC, and AS whole blood. (**I**) Venn diagram showing the overlap of *STAT3^+^* enriched GO and KEGG pathways across CD, UC, and AS whole blood.

We integrated machine learning and MR analyses to identify core genes associated with CD, UC, and AS. The machine learning models revealed that *STAT3* and *HYOU1* serve as key genes that differentiate diseased samples from normal controls. Subsequent exploratory MR analysis identified disease-specific associations between genetically predicted STAT3 expression and the three conditions, with positive associations for CD and UC and an inverse association for AS. Notably, *STAT3* exhibited a strong association with both CD and UC. Compared to normal controls, *STAT3* expression was reduced in samples from CD and UC patients, whereas it was elevated in whole blood samples from AS patients. We categorized all CD, UC, and AS samples into *STAT3*^+^ and *STAT3*^−^ subgroups based on the median expression level of *STAT3*. In the *STAT3*^+^ samples, we identified 90 upregulated genes and 409 downregulated genes in CD (Figure 6A), 168 upregulated genes and 36 downregulated genes in UC (Figure 6B), and 360 upregulated genes and 196 downregulated genes in AS whole blood samples (Figure 6C).

Distinct pathway signatures were revealed between the *STAT3*^+^ and *STAT3*^−^ groups using gene set variation analysis (GSVA). In CD, the *STAT3*^+^ group exhibited enrichment in amine catabolic process, amino acid betaine metabolic process, and anterograde axonal transport of mitochondrion pathways, while the *STAT3*^−^ group showed enrichment in antigen processing and presentation via MHC class II, amacrine cell differentiation, and amylin receptor signaling pathway pathways (Figure 6D). In UC, the *STAT3*^+^ group displayed enrichment in acetyl-CoA biosynthesis from pyruvate, aerobic respiration, and acetyl-CoA biosynthesis, whereas the *STAT3*^−^ group was enriched in angiogenesis in coronary vascular morphogenesis, antibody-dependent cellular cytotoxicity, and aromatic amino acid transport (Figure 6E). Analysis of AS whole blood samples indicated that the *STAT3*^+^ group exhibited enrichment in AMP biosynthesis, 2-oxoglutarate metabolic process, and aspartate metabolic process, while the *STAT3*^−^ group showed enrichment in cysteine-type endopeptidase activation, protein kinase B (Akt) activation, and amyloid-beta clearance via transcytosis (Figure 6F). Remarkably, the oxoglutarate metabolic process was consistently upregulated in the *STAT3*^+^ group in both CD and AS samples. Furthermore, multiple biological pathways were enriched and upregulated in *STAT3*^+^ samples from UC and AS, including aerobic respiration, amino acid betaine metabolism, alditol biosynthesis, amino acid betaine biosynthesis, 2Fe-2S cluster assembly, acetyl-CoA biosynthesis, and acetyl-CoA metabolism (Figure 6I).

### 3.7. Drug Prediction and Molecular Docking Analysis

Transcriptome-based drug-response prediction was performed separately in the CD, UC, and AS datasets. Correlation heatmaps illustrated the relationships between disease-associated NRG expression and predicted drug-response scores in the three disease groups (Supplementary Figure S5A–C). Comparison of the candidate sets identified four agents shared between AS and at least one IBD dataset: ciclosporin, BCL-LZH-4, BRD-K79669418, and CID-5951923 (Supplementary Figure S5D). Ciclosporin is an established pharmacological agent, whereas BCL-LZH-4, BRD-K79669418, and CID-5951923 were regarded as experimental compounds in the present analysis. Exploratory molecular docking against STAT3 generated valid poses for BCL-LZH-4 and CID-5951923, with DOCK Grid Scores of −35.321896 and −28.361128, respectively. Docking of BRD-K79669418 did not generate a completed ligand-growth pose and was therefore excluded from score-based interpretation. Although BCL-LZH-4 exhibited the more negative DOCK Grid Score of −35.321896 against the signal transducer and activator of STAT3, no conventional hydrogen-bond interaction was identified under the visualization criteria used, and it was therefore not selected for representative visualization. In contrast, CID-5951923 achieved a DOCK Grid Score of −28.361128 against STAT3. Analysis of the predicted binding pose indicated that CID-5951923 was located within a pocket surrounded by THR526, THR527, GLU530, GLY536, VAL537, TYR539, ILE589, SER590, LYS591, GLU612, and ARG609. A potential hydrogen-bond interaction was observed with ARG609 at an interatomic distance of approximately 2.8 Å, together with additional close contacts with the surrounding STAT3 residues at distances of approximately 3.1–3.5 Å. CID-5951923 was therefore selected as the representative predicted STAT3–ligand interaction model (Supplementary Figure S5E). These computational findings prioritize BCL-LZH-4 and CID-5951923 for subsequent biochemical and experimental evaluation.

### 3.8. Single-Cell Transcriptomic Analysis Identified Tissue- and Cell-Type-Specific STAT3 Expression Patterns

Single-cell RNA sequencing (scRNA-seq) was performed to characterize cell-type-specific *STAT3* mRNA expression patterns in the analyzed IBD colonic and AS peripheral-blood datasets. Two scRNA-seq datasets, GSE214695 and GSE232131, were utilized in this study. GSE214695 included six normal colon samples, six samples from patients with CD, and six samples from patients with UC. In contrast, GSE232131 comprised six peripheral blood mononuclear-cell samples from patients with AS, including three unstimulated samples and three ex vivo immune-stimulated samples. Given the lack of suitable scRNA-seq datasets for AS tissue, peripheral blood samples from AS patients were analyzed. To ensure analytical consistency, consensus cell types were retained across CD, UC, and AS whole blood samples, including T cells, NK cells, monocytes, dendritic cells, B cells, plasma cells, stromal cells, epithelial cells, and endothelial cells, but their proportions were not directly compared across the colonic and peripheral-blood datasets. Notably, platelets were identified as a specific cell subtype associated with AS. Data preprocessing involved selecting the top 1500 genes exhibiting the highest intercellular variability. PCA was employed for initial dimensionality reduction, followed by uniform manifold approximation and projection (UMAP) for cell clustering.

**Figure 7:**
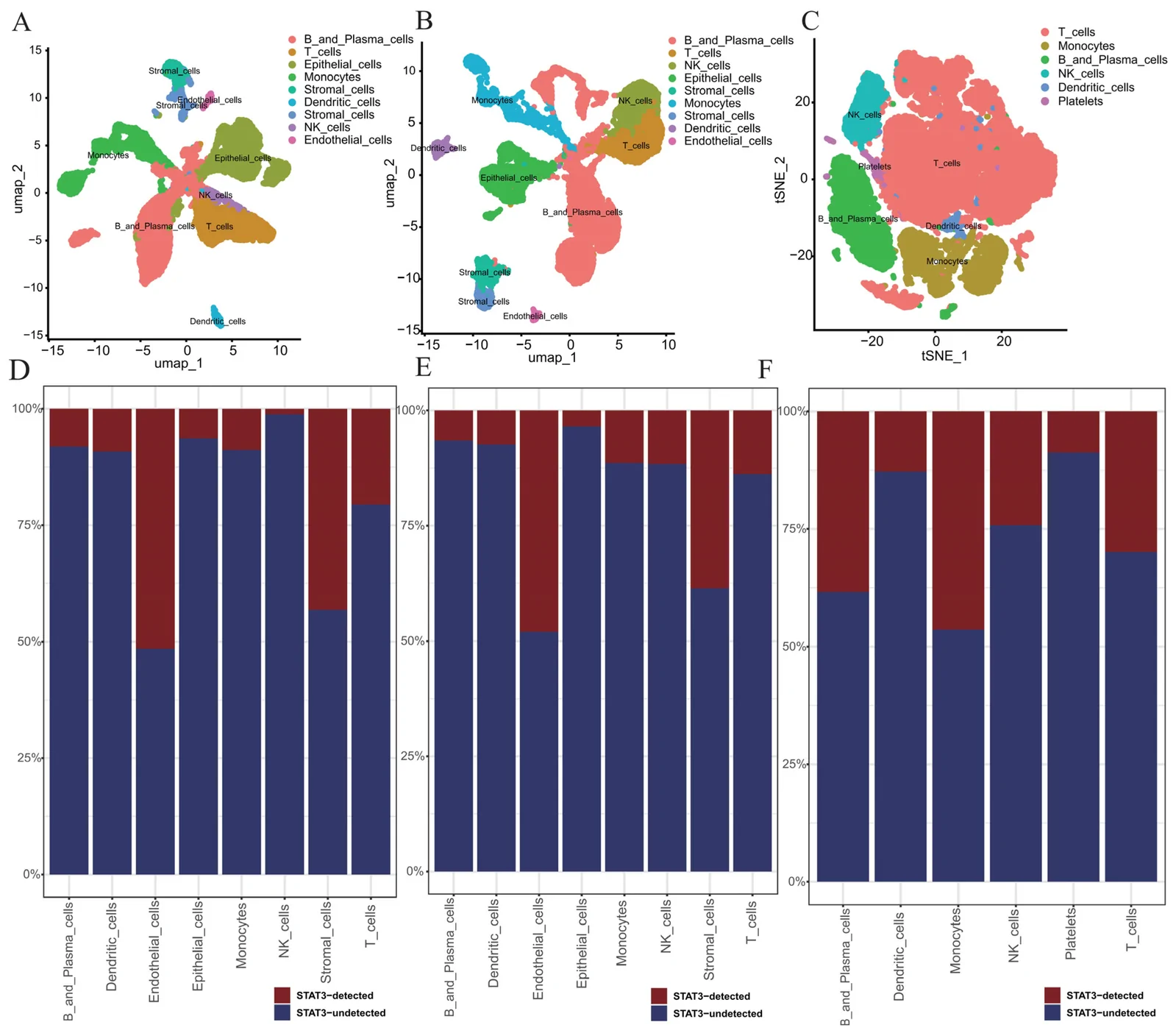
Single-cell transcriptomic analysis of STAT3 across CD, UC, and AS. (**A**,**B**) UMAP plots showing the annotated cell populations in the CD and UC colonic datasets, respectively. (**C**) t-SNE plot showing the annotated cell populations in the AS peripheral-blood dataset. (**D**–**F**) Bar plots showing the proportions of cells with detected and undetected *STAT3* expression within each annotated cell type. *STAT3*-detected cells were defined as cells with normalized *STAT3* expression > 0, whereas *STAT3*-undetected cells had a normalized expression value of 0. These panels illustrate dataset- and cell-type-specific patterns of *STAT3* transcript detection.

We initially analyzed IBD scRNA-seq data from GSE214695, annotating core cell lineages in CD samples as B and plasma cells, T cells, and epithelial cells. The distribution of cell types in CD and healthy controls is illustrated in Figure 7A, while the landscape of UC and matched controls is depicted in Figure 7B. The GSE232131 dataset contained T cells, monocytes, B and plasma cells, NK cells, dendritic cells, and platelets (Figure 7C). Bar plots were used to summarize the proportions of cells with detected and undetected *STAT3* expression within each annotated cell type. In the IBD colonic dataset, detected *STAT3* expression was observed particularly in endothelial and stromal cells (Figure 7D,E). In the AS peripheral-blood dataset, monocytes represented a major population exhibiting detected *STAT3* expression, while *STAT3* expression was also observed in NK cells, B and plasma cells, and platelets (Figure 7F). Together, these results delineate dataset- and cell-type-specific *STAT3* mRNA expression patterns and provide a cellular framework for subsequent functional investigation. Bubble plots were generated to assess the accuracy of cell annotation and to identify the expression of canonical marker genes (Supplementary Figure S6A–C). In samples from patients with CD, endothelial cells, stromal cells, and monocytes exhibited significant expression of their respective signature genes (Supplementary Figure S6A). In contrast, endothelial, stromal, and epithelial cells in the UC dataset expressed their corresponding canonical markers, supporting the cell-type annotations (Supplementary Figure S6B). The annotated cell types in the AS peripheral-blood dataset included T cells, NK cells, monocytes, dendritic cells, B cells, and plasma cells, with canonical marker expression supporting the identification of B cells, plasma cells, and monocytes (Supplementary Figure S6C).

Cell-type-specific expression patterns of the core NRGs were subsequently examined separately within the CD, UC, and AS datasets (Supplementary Figure S7A–C). In CD, *STAT3* expression remained relatively stable across the annotated cell types (Supplementary Figure S7A). However, *IL1B* showed comparatively higher expression in B and plasma cells, monocytes, epithelial cells, and endothelial cells, highlighting its cell-type-specific transcriptional distribution in the CD dataset. In UC, *STAT3* and *IL1B* exhibited comparatively higher expression in monocytes and lower expression in endothelial cells (Supplementary Figure S7B). In the AS peripheral-blood dataset, *STAT3* expression was more prominent in NK cells, B and plasma cells, and platelets, indicating a cell-type-specific expression pattern (Supplementary Figure S7C). Overall, *STAT3* showed limited expression differences across cell types in CD and UC but more pronounced expression patterns in several annotated cell populations in the AS dataset. *IL1B* also exhibited expression across multiple cell populations in the three analyzed datasets.

The expression patterns of 32 Co-DE-NRGs across annotated cell types were examined (Supplementary Figure S8A–C). In CD and UC, *CALR*, *CD63*, and *CYBA* exhibited widespread expression in most cell populations, except in NK cells. In contrast, *STAT3* expression was predominantly restricted to endothelial and stromal cells (Supplementary Figure S8A,B). In the AS peripheral-blood dataset, *CALR*, *CD63*, and *CYBA* were expressed across multiple cell types, including NK cells and monocytes, whereas dendritic cells exhibited comparatively limited expression differences. *STAT3* showed a distinct cell-type-specific expression pattern, with monocytes representing a major population exhibiting detected *STAT3* expression (Supplementary Figure S8C). Together, these findings highlight the cellular heterogeneity of NRG expression in AS peripheral blood and prioritize *STAT3* expression in monocytes for subsequent functional investigation.

## 4. Discussion

Accumulating clinical and preclinical evidence has established a strong bidirectional comorbid relationship between IBD and AS. Although TRPM4-dependent NECSO provides a potential biological framework for investigating sodium-overload-related cellular injury [50], direct evidence supporting its involvement in IBD-AS comorbidity is currently lacking. In the present study, we characterized the expression profiles of NRGs in IBD and AS and identified shared transcriptional alterations that warrant further functional investigation.

Our differential expression analysis identified 32 overlapping Co-DE-NRGs shared by IBD and AS, providing a molecular basis for exploring the potential links between these two autoimmune diseases. Previous transcriptomic studies have used exploratory differential-expression screening to prioritize biologically relevant candidate genes [48,49]. Following this exploratory framework, we initially applied the nominal criteria of *p* < 0.05 and |log2FC| > 1 and identified 32 Co-DE-NRGs shared between the IBD and AS datasets. Because multiple NRGs were tested within each dataset, we additionally applied the Benjamini–Hochberg procedure to evaluate the robustness of the observed associations. *STAT3* remained significantly differentially expressed after multiple-testing correction in the CD and UC datasets but not in the AS whole-blood dataset (Supplementary Table S2). Therefore, the 32 Co-DE-NRGs identified using the nominal criteria should be regarded as an exploratory candidate set rather than as uniformly FDR-confirmed differentially expressed genes. Further validation in independent datasets and disease-relevant experimental models is required.

Functional enrichment and interaction analysis of the 32 Co-DE-NRGs revealed a tightly connected protein–protein interaction network enriched with core NECSO regulators, including PTEN, BECN1, MYC, STAT3, and IL1B. Co-expression patterns of these shared genes differed significantly between healthy controls and patients, with the most prominent transcriptional perturbation observed in AS samples. Immune infiltration correlation analysis further demonstrated that altered expression of Co-DE-NRGs reshaped the immune microenvironment of lesion tissues. These genes were negatively correlated with the infiltration of resting mast cells, CD8^+^ T cells, and plasma cells, but positively correlated with neutrophils and myeloid inflammatory cells. Collectively, these findings suggest that the identified Co-DE-NRGs drive IBD and AS progression by remodeling immune cell infiltration landscapes.

The integrated analyses provided three distinct but complementary forms of evidence regarding *STAT3*. At the genetic level, exploratory MR showed positive associations of genetically predicted *STAT3* expression with CD and UC but an inverse association with AS, indicating a disease-specific and context-dependent pattern of genetic association. At the transcriptional level, bulk and single-cell analyses identified disease- and cell-type-specific patterns of *STAT3* mRNA expression and associations with immune-cell composition, supporting its relevance to immune-related molecular alterations in IBD and AS. At the discriminatory level, *STAT3* was retained as a candidate feature across the CD, UC, and AS machine-learning analyses, with individual AUCs of 0.894, 0.752, and 0.635, respectively. These results indicate comparatively strong discrimination in CD, moderate discrimination in UC, and more limited discrimination in AS. Collectively, the genetic, transcriptional, and discriminatory evidence consistently prioritized *STAT3* as a cross-disease candidate gene while also demonstrating that its direction and performance varied among the three diseases. Further functional and independent clinical validation is required to define its disease-specific biological roles and translational value.

STAT3 is a core hub molecule regulating the initiation and progression of autoimmune diseases, and its dysfunction is a key driver of multiple autoimmune disorders [51]. Numerous studies have established that phosphorylated STAT3 serves as a principal pro-inflammatory mediator in IBD. In the joints, the inflammatory activation of synovial Th17 cells and macrophages also depends on STAT3 signaling. The activity of STAT3 is tightly regulated by reversible palmitoylation cycles, with the enzyme ZDHHC7 facilitating STAT3 palmitoylation. Palmitoylated STAT3 exhibits an increased affinity for the IL-6R/IL-23R membrane receptor complex, significantly enhancing JAK-mediated tyrosine phosphorylation, nuclear translocation, and transcriptional activity of STAT3. Conversely, APT2 induces depalmitoylation, which releases STAT3 from the cell membrane and reverts it to an inactive cytoplasmic state. Together, these two enzymes sustain a stable modification cycle. Disruption of this equilibrium—such as diminished APT2 activity or elevated ZDHHC7 expression—results in persistent STAT3 palmitoylation and excessive phosphorylation-driven activation [52]. We hypothesize that this mechanism in intestinal T cells could drive systemic Th17 migration to the sacroiliac joints, linking gut inflammation to joint damage in AS. While no published studies have directly connected STAT3 palmitoylation or phosphorylation to AS. Our analysis suggests a plausible inference: disruption of palmitoylation or phosphorylation homeostasis in intestinal T cells leads to a systemic overexpansion of Th17 cells. These cells subsequently migrate to and infiltrate the sacroiliac joints, ultimately causing the local inflammatory damage that is characteristic of AS. We also identified a highly synergistic co-expression network comprising *STAT3*, *STAT1*, *GBP4*, *GBP2*, *NAMPT*, *STX11*, *GBP5*, *ANKRD22*, *BASP1*, *PLEK*, *FPR2*, *VNN2*, *IL1B*, *FCGR2A*, *FPR1*, and *MMP9*. These co-expressing genes may act as a regulator of *STAT3* in the occurrence of NECSO in IBD and AS. Nevertheless, these in silico findings need further verification through in vivo and in vitro functional experiments.

In addition to *STAT3*, our integrative analysis identified several other core NECSO regulators with disease-relevant roles in both IBD and AS. *IL1B* is involved in diverse biological processes, such as NECSO activation, maintenance of cellular homeostasis, and remodeling of central neural circuits [53]. In the context of IBD, dysregulated *IL1B* expression triggers NECSO activation, compromises intestinal barrier integrity, and disrupts local immune balance. In AS, abnormal *IL1B* expression hinders neural plasticity remodeling and stress regulation pathways [54]. These disruptions in pathways help explain the high co-occurrence of IBD and AS, although the mechanistic links are currently theoretical and necessitate direct validation. Moreover, mutations in *CYBA* and other NADPH oxidase genes inhibit ROS production in neutrophils of pediatric patients, leading to disturbances in glucose metabolism and antibacterial defenses. The deficiency of neutrophil ROS is closely associated with these genetic alterations. *CYBA* collectively governs the dysfunction of the NADPH oxidase-ROS pathway, influencing the development of complications, disease advancement, and antibacterial immunity in Crohn’s disease, thus contributing to the pathogenesis of IBD [55]. IBD results from interactions between genes and the environment. ADAR, an A-to-I RNA-editing enzyme, plays a crucial role in maintaining intestinal homeostasis. Reduced ADAR levels in intestinal crypts contribute to the development of IBD. Inhibiting ADAR either genetically or pharmacologically triggers the onset of spontaneous intestinal inflammation in mice. The depletion of ADAR leads to an accumulation of double-stranded RNA (dsRNA) and endogenous retroviruses (ERVs), activating the MDA5-mediated dsRNA sensing pathway and the JAK-STAT cascades, ultimately disrupting intestinal homeostasis [56].

From a clinical perspective, the present findings do not yet support the use of STAT3 or the other Co-DE-NRGs for diagnosis, treatment selection, or therapeutic monitoring. Patients with coexisting or suspected IBD and axSpA should continue to receive coordinated assessment by gastroenterologists and rheumatologists, with management guided by intestinal and musculoskeletal disease activity, established biomarkers, imaging findings, treatment history, and safety considerations. Consistent with current recommendations, the presence of active IBD should be considered when selecting therapy for axSpA. The genes and compounds prioritized in this study should therefore be regarded as candidates for subsequent functional and prospective clinical evaluation rather than as immediately applicable therapeutic recommendations.

Several limitations of this study should be acknowledged. First, the IBD datasets were derived from colonic biopsies, whereas the AS datasets were derived from peripheral whole blood. Although disease-control comparisons were performed separately within each tissue type, the subsequent identification of overlapping genes could not fully distinguish shared disease-related alterations from tissue-specific expression patterns or differences in cellular composition. Whole-blood analysis may capture systemic immune alterations associated with AS but cannot reproduce the local molecular environment of joint or entheseal tissues. Moreover, although two AS datasets were combined to increase the available sample size, the resulting sample size remained relatively small and did not provide fully independent external validation. Future studies using matched blood, intestinal, and musculoskeletal tissue samples are required to assess the tissue specificity and reproducibility of the identified associations.

The single-cell analysis was subject to similar tissue-related and sample-size limitations. The IBD single-cell data were derived from colonic tissue, whereas the AS data were derived from peripheral blood and included only a limited number of samples. Therefore, cell-type proportions and *STAT3* expression patterns were interpreted within their respective datasets rather than directly compared across different tissue compartments. Furthermore, because zero values in sparse scRNA-seq data may reflect either low transcript abundance or limited transcript capture, the *STAT3*-detected and *STAT3*-undetected categories should be interpreted as descriptive summaries of transcript detection. Larger tissue-matched single-cell datasets, together with functional measurements of STAT3 protein abundance, phosphorylation, nuclear translocation, and transcriptional activity, will be required to determine the biological significance of the observed cell-type-specific expression patterns. Second, the 32 Co-DE-NRGs shared between IBD and AS were identified primarily through bioinformatic analyses, and their biological functions and molecular mechanisms remain to be experimentally validated. Future studies should employ cellular and animal models of colitis, AS, and IBD–AS comorbidity to examine the tissue-specific expression and functional roles of core genes such as *STAT3*. These investigations should incorporate qPCR and Western blotting, together with functional assays, to determine whether the identified genes contribute to the pathological processes shared by IBD and AS. Third, although GEO accession-defined cohorts were excluded from supervised feature selection and model fitting, the model-development and held-out datasets were jointly included in the unsupervised ComBat harmonization step. In addition, held-out-cohort performance contributed to the descriptive comparison of candidate modeling pipelines. Therefore, the current results should be regarded as a cohort-held-out exploratory evaluation rather than fully independent external validation. The reduced performance in the held-out cohorts, particularly the limited specificity of the AS model, further indicates that these models are not yet suitable for clinical application. Fully independent validation will require preprocessing, feature selection, model optimization, and threshold determination to be completed without access to the external cohorts, followed by prospective evaluation in larger and clinically well-characterized populations. Fourth, the transcriptomic datasets analyzed in this study did not include direct measurements of TRPM4 activity, intracellular Na^+^ accumulation, osmotic swelling, membrane rupture, or other cellular features required to identify NECSO. Therefore, the observed expression patterns of NRGs do not demonstrate that NECSO occurs in IBD or AS or that it mediates the association between the two diseases. Establishing the involvement of NECSO will require direct functional validation using disease-relevant cellular and animal models. Finally, this study was not designed to evaluate established clinical biomarkers or treatment-response markers. Candidate selection was restricted to a predefined NRG-related gene set and required shared differential expression across the CD, UC, and AS datasets. Consequently, established markers outside this analytical scope, including C-reactive protein and fecal calprotectin in IBD and C-reactive protein and erythrocyte sedimentation rate in axSpA, were not expected to be retained among the 32 Co-DE-NRGs. Although several prioritized genes, including JAK1, STAT3, and IL1B, intersect with recognized inflammatory pathways, they should be regarded as exploratory candidates rather than replacements for clinically validated biomarkers. Their diagnostic and treatment-response value requires evaluation in prospective, clinically characterized cohorts.

## 5. Conclusions

In conclusion, we employed a combination of bulk transcriptomics, single-cell RNA sequencing, drug–target interaction analysis, molecular docking, machine learning, and exploratory MR analysis to characterize shared NRG-related molecular signatures in IBD and AS. Multiple complementary analyses prioritized *STAT3* as a cross-disease candidate gene with disease-, tissue-, and cell-type-dependent patterns. Single-cell analysis delineated dataset- and cell-type-specific *STAT3* mRNA expression, with monocytes representing a prominent population exhibiting detected *STAT3* expression in the AS peripheral-blood dataset. Exploratory MR analysis identified positive associations between genetically predicted *STAT3* expression and CD and UC but an inverse association with AS, indicating a disease-specific rather than uniform genetic association pattern. Compound screening and molecular docking further prioritized candidate compounds for subsequent experimental evaluation. Overall, these findings provide a hypothesis-generating framework for investigating shared NRG-related transcriptional alterations in intestinal and joint inflammatory diseases. Further functional investigations using disease-relevant experimental models are necessary to determine the potential contribution of NECSO to the association between intestinal and joint inflammation and to assess the biological and translational significance of the identified candidate genes.

## Data Availability

All transcriptomic datasets analyzed in this study are publicly available from the Gene Expression Omnibus under the accession numbers reported in the Methods. The eQTL and GWAS summary data used for the exploratory MR analysis are available through the IEU OpenGWAS database. Supporting analytical outputs are provided in the Supplementary Figures and Tables. The machine-learning analysis scripts, supporting functions, software dependencies, and execution instructions are included in Supplementary_Code.zip accompanying this article.

## Supplementary Materials

The following supporting information can be downloaded at: https://www.mdpi.com/article/10.3390/genes17080938/s1. Figure S1: Principal component analysis plots showing sample distributions before and after batch correction in the CD, UC, and AS datasets. Panels A, C, and E show the distributions before batch correction for CD, UC, and AS, respectively, whereas panels B, D, and F show the corresponding distributions after batch correction; Figure S2: Systematic comparison of machine-learning pipelines for CD, UC, and AS. Heatmaps showing the AUC values of the evaluated machine-learning pipelines in the model-development and held-out cohorts for (A) CD, (B) UC, and (C) AS. After excluding pipelines that retained no more than five features, 102, 113, and 59 pipelines were evaluated for CD, UC, and AS, respectively. The rightmost column presents the summary AUC used for exploratory model comparison. AUC, area under the receiver operating characteristic curve; CD, Crohn’s disease; UC, ulcerative colitis; AS, ankylosing spondylitis; Figure S3: Cohort-held-out evaluation of the representative machine-learning models for CD, UC, and AS. (A,B) ROC curves of the CD XGBoost model in the model-development cohort and the held-out GSE179285 cohort, respectively. (C,D) Confusion matrices of the CD model in the model-development and held-out cohorts, respectively. (E,F) ROC curves of the UC random-forest model in the model-development cohort and the held-out GSE179285 cohort, respectively. (G,H) Confusion matrices of the UC model in the model-development and held-out cohorts, respectively. (I,J) ROC curves of the AS Stepglm[backward]+RF model in the model-development cohort and the held-out GSE25101 cohort, respectively. (K,L) Confusion matrices of the AS model in the model-development and held-out cohorts, respectively; Figure S4: Differential-expression characteristics and individual discriminatory performance of genes retained by the representative models. (A) Differential-expression distribution of the displayed genes retained by the CD XGBoost model. (B) ROC curves evaluating the individual discriminatory performance of these genes in the CD model-development cohort. (C) Differential-expression distribution of the displayed genes retained by the UC random-forest model. (D) ROC curves evaluating their individual discriminatory performance in the UC model-development cohort. (E) Differential-expression distribution of the genes retained by the AS Stepglm[backward]+RF model. (F) ROC curves evaluating their individual discriminatory performance in the AS model-development cohort. The numbered genes in panels A, C, and E are ordered according to their adjusted *p* values and do not represent model-derived feature-importance rankings. AUC, area under the curve; CD, Crohn’s disease; RF, random forest; ROC, receiver operating characteristic curve; UC, ulcerative colitis; AS, ankylosing spondylitis; Figure S5: Transcriptome-based drug-response prediction and molecular docking of candidate compounds with STAT3. (A–C) Heatmaps showing Pearson correlations between the expression levels of disease-associated Na^+^ overload-related genes and the predicted CTRP2 drug-response scores in the (A) CD, (B) UC, and (C) AS datasets. Candidate compounds were prioritized using coverage thresholds of 90% for CD, 80% for UC, and 60% for AS. (D) Overlap of the prioritized compounds across the three disease-specific analyses. Four compounds—ciclosporin, BCL-LZH-4, BRD-K79669418, and CID-5951923—were shared between AS and at least one IBD dataset. (E) Representative predicted binding pose of CID-5951923 within the STAT3 protein structure (PDB ID: 6TLC). The ligand was positioned in a pocket surrounded by THR526, THR527, GLU530, GLY536, VAL537, TYR539, ILE589, SER590, LYS591, GLU612, and ARG609. A potential hydrogen-bond interaction with ARG609 was observed at an interatomic distance of approximately 2.8 Å. The predicted STAT3–CID-5951923 complex yielded a DOCK Grid Score of −28.361128. AS, ankylosing spondylitis; CD, Crohn’s disease; CTRP2, Cancer Therapeutics Response Portal version 2; IBD, inflammatory bowel disease; NRG, Na^+^ overload-related gene; STAT3, signal transducer and activator of transcription 3; UC, ulcerative colitis; Figure S6: Bubble plots showing canonical marker-gene expression across annotated cell types in the (A) CD, (B) UC, and (C) AS datasets; Figure S7: Heatmaps showing cell-type-specific differential-expression patterns of the 32 Co-DE-NRGs in the (A) CD, (B) UC, and (C) AS datasets; Figure S8: Heatmaps showing the expression ratios of the 32 Co-DE-NRGs across annotated cell types in the (A) CD, (B) UC, and (C) AS datasets; Table S1: Complete GeneCards search results for Na^+^ overload-induced necrotic death; Table S2: Nominal *p*-values and FDR-adjusted q-values for differential expression between normal and diseased samples in CD, UC, and AS whole blood; Table S3: Performance of representative machine-learning models in model-development and held-out GEO cohorts; Table S4: Candidate compounds prioritized by transcriptome-based drug-response prediction and exploratory molecular docking against STAT3.

## Abbreviations

The following abbreviations are used in this manuscript:

AS	Ankylosing Spondylitis
IBD	Inflammatory Bowel Disease
CD	Crohn’s disease
UC	Ulcerative colitis
TRPM4	Transient receptor potential cation channel subfamily M, member 4
NC1	Necrocide 1
SpA	Spondyloarthritis
axSpA	Axial spondyloarthritis
IMIDs	Immune-Mediated Inflammatory Diseases
RCD	Regulated Cell Death
NECSO	Na^+^ by sodium overload
NRGs	Na^+^ overload-related genes
DE-NRGs	Differentially Expressed Na^+^ overload-related genes
Co-DE-NRGs	Common Differentially Expressed NRGs
PCA	Principal Component Analysis
GEO	Gene Expression Omnibus
PPI	Protein–protein Interaction
SVM	Support Vector Machine
RF	Random Forest
GLM	Generalized Linear Model
KNN	k-Nearest Neighbors
ROC	Receiver Operating Characteristic
AUC	Area Under the Curve
MR	Mendelian Randomization
GSVA	Gene Set Variation Analysis
GWAS	Genome-wide Association Studies
IVs	Instrumental variables
IVW	Inverse-variance weighted
scRNA-seq	Single-cell RNA sequencing
UMAP	Uniform Manifold Approximation and Projection
