# Supplementary material for: Integrated Transcriptomic and Machine-Learning Analyses Identify Shared Na+ Overload-Related Gene Signatures in Inflammatory Bowel Disease and Ankylosing Spondylitis
Source: Genes (Basel). 2026 Aug 11;17(8):938. doi: 10.3390/genes17080938 (PMC13512338; doi:10.3390/genes17080938)
Supplement: Supplementary file 1 [file genes-17-00938-s001.zip › Supplementary Figures and Tables.pdf]

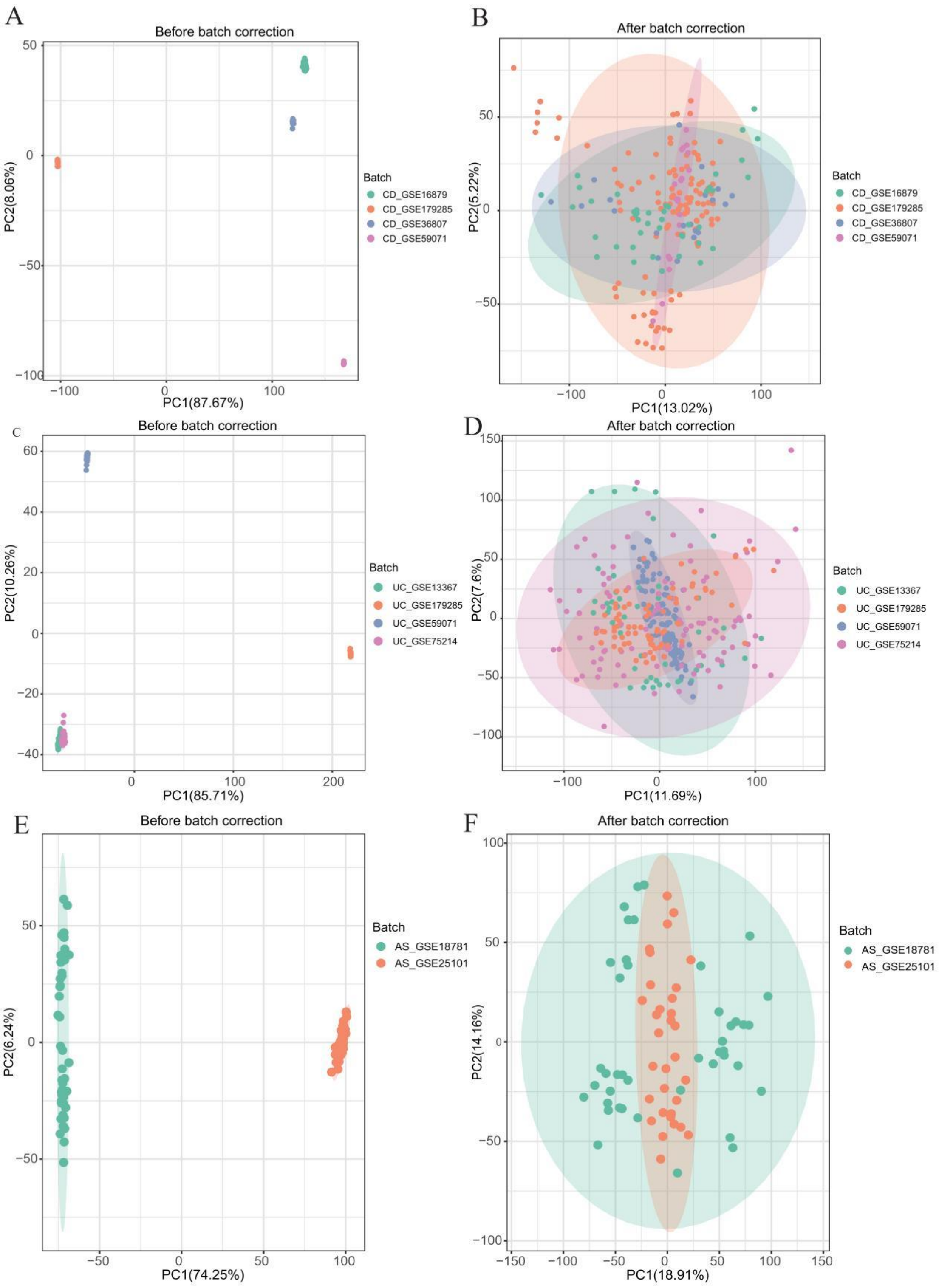

Supplementary Figure S1. Principal component analysis plots showing sample distributions before and after batch correction in the CD, UC, and AS datasets. Panels A, C, and E show the distributions before batch correction for CD, UC, and AS, respectively, whereas panels B, D, and F show the corresponding distributions after batch correction.

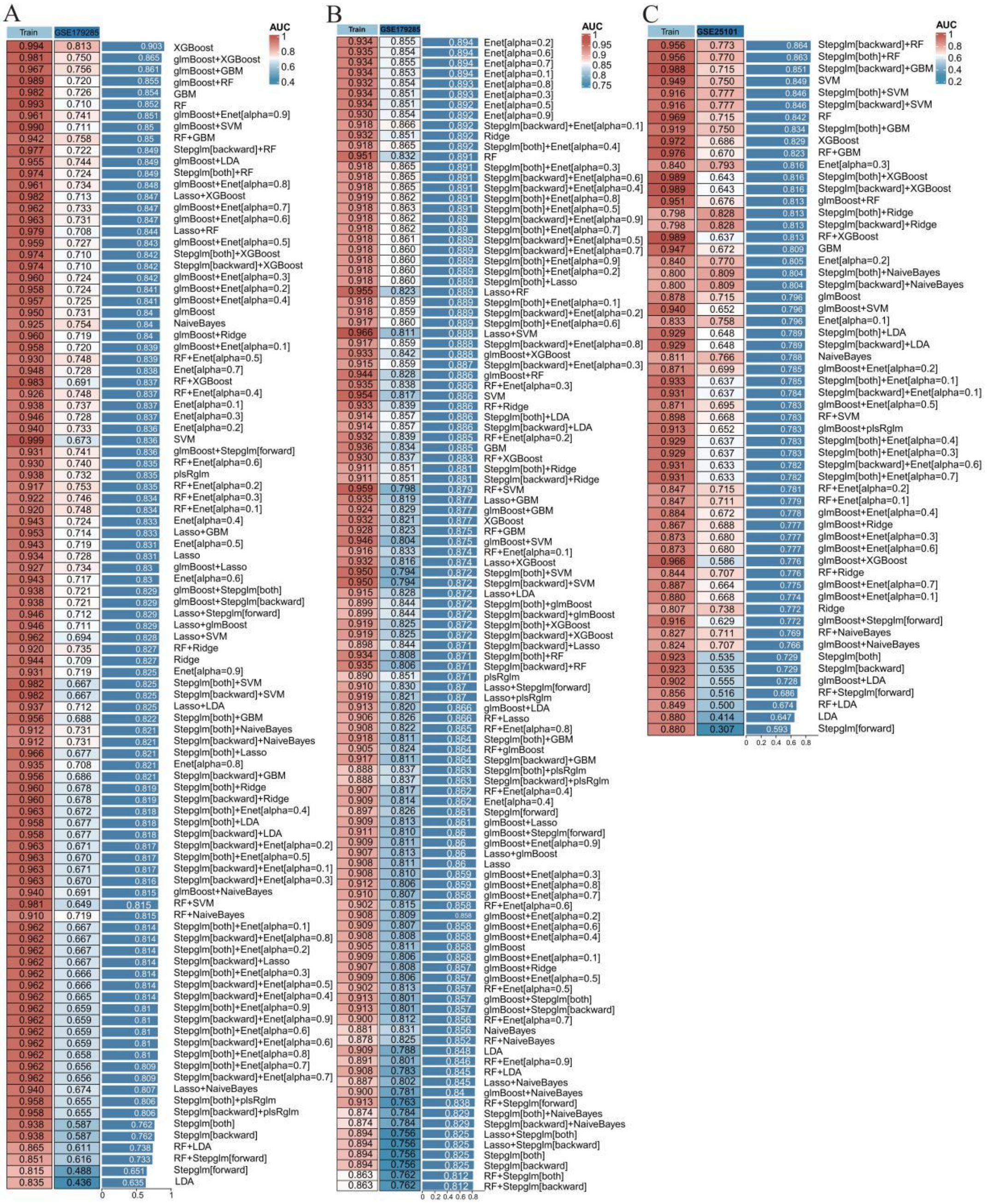

Supplementary Figure S2. Systematic comparison of machine-learning pipelines for CD, UC, and AS. Heatmaps showing the AUC values of the evaluated machine-learning pipelines in the model-development and held-out cohorts for (A) CD, (B) UC, and (C) AS. After excluding pipelines that retained no more than five features, 102, 113, and 59 pipelines were evaluated for CD, UC, and AS, respectively. The rightmost column presents the summary AUC used for exploratory model comparison. AUC, area under the receiver operating characteristic curve; CD, Crohn's disease; UC, ulcerative colitis; AS, ankylosing spondylitis.

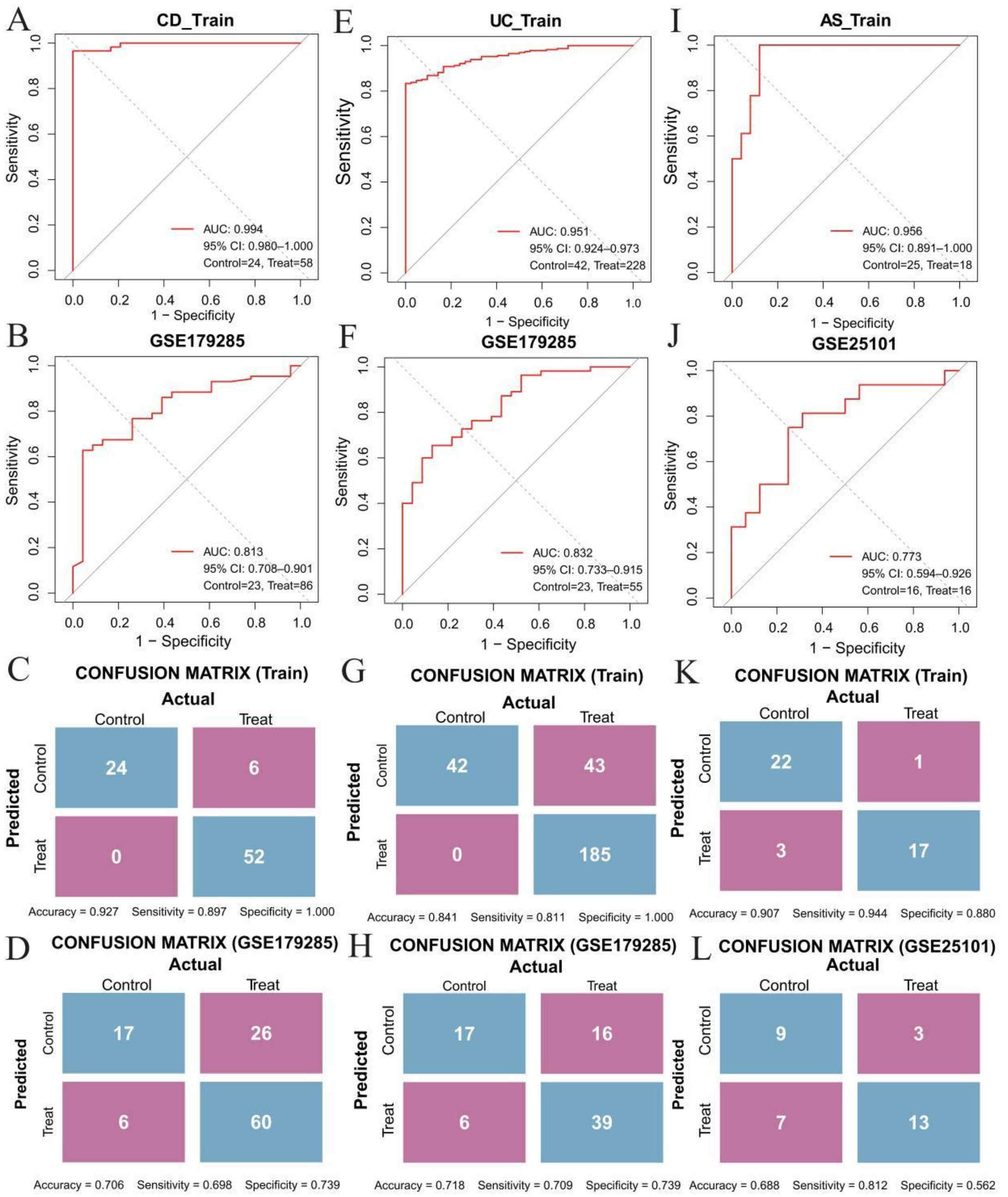

Supplementary Figure S3. Cohort-held-out evaluation of the representative machine-learning models for CD, UC, and AS. (A,B) ROC curves of the CD XGBoost model in the model-development cohort and the held-out GSE179285 cohort, respectively. (C,D) Confusion matrices of the CD model in the model-development and held-out cohorts, respectively. (E,F) ROC curves of the UC random-forest model in the model-development cohort and the held-out GSE179285 cohort, respectively. (G,H) Confusion matrices of the UC model in the model-development and held-out cohorts, respectively. (I,J) ROC curves of the AS Stepglm[backward]+RF model in the model-development cohort and the held-out GSE25101 cohort, respectively. (K,L) Confusion matrices of the AS model in the model-development and held-out cohorts, respectively.

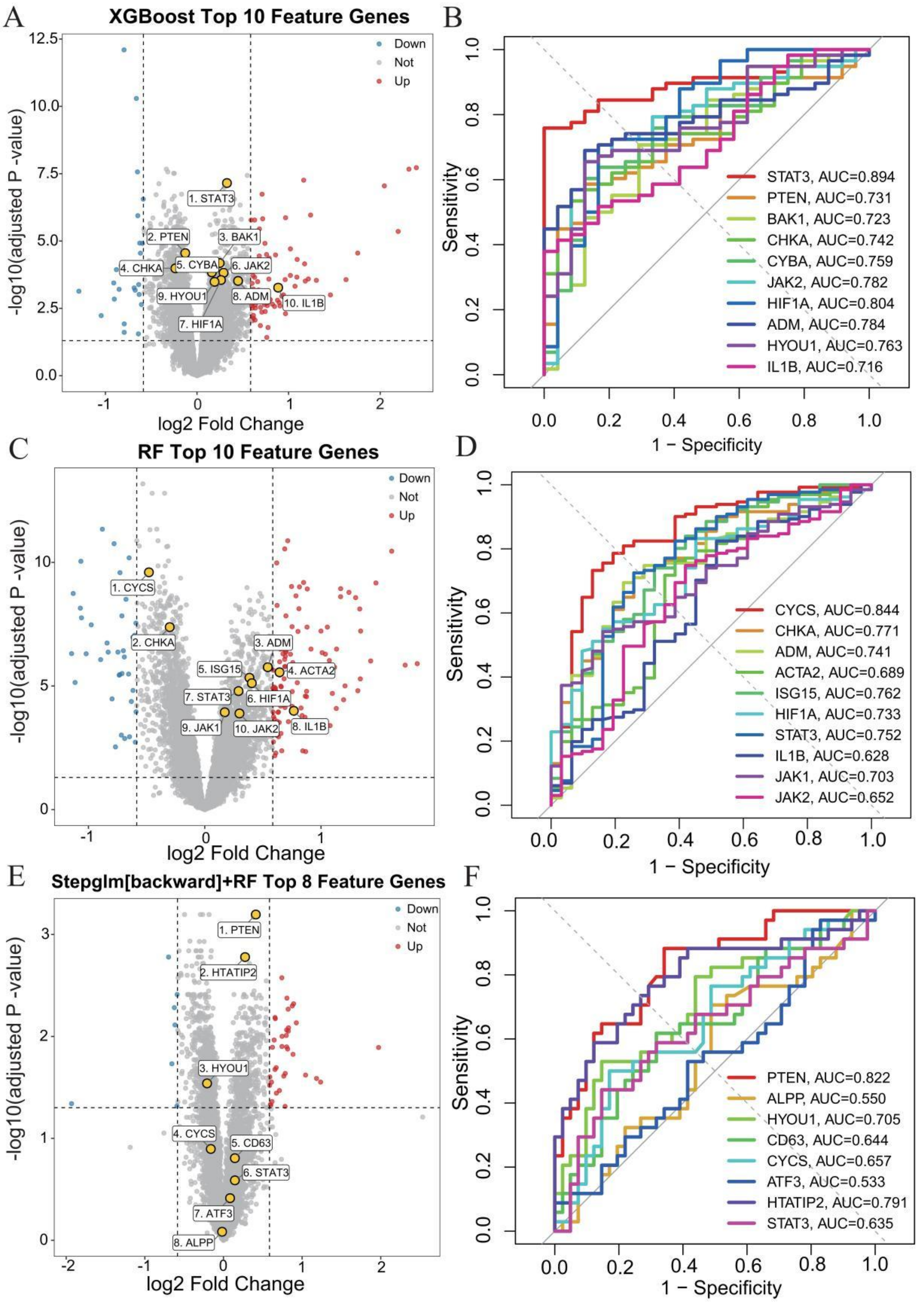

Supplementary Figure S4. Differential-expression characteristics and individual discriminatory performance of genes retained by the representative models. (A) Differential-expression distribution of the displayed genes retained by the CD XGBoost model. (B) ROC curves evaluating the individual discriminatory performance of these genes in the CD model-development cohort. (C) Differential-expression distribution of the displayed genes retained by the UC random-forest model. (D) ROC curves evaluating their individual discriminatory performance in

the UC model-development cohort. (E) Differential-expression distribution of the genes retained by the AS Stepglm[backward]+RF model. (F) ROC curves evaluating their individual discriminatory performance in the AS model-development cohort. The numbered genes in panels A, C, and E are ordered according to their adjusted p values and do not represent model-derived feature-importance rankings. AUC, area under the curve; CD, Crohn's disease; RF, random forest; ROC, receiver operating characteristic curve; UC, ulcerative colitis; AS, ankylosing spondylitis.

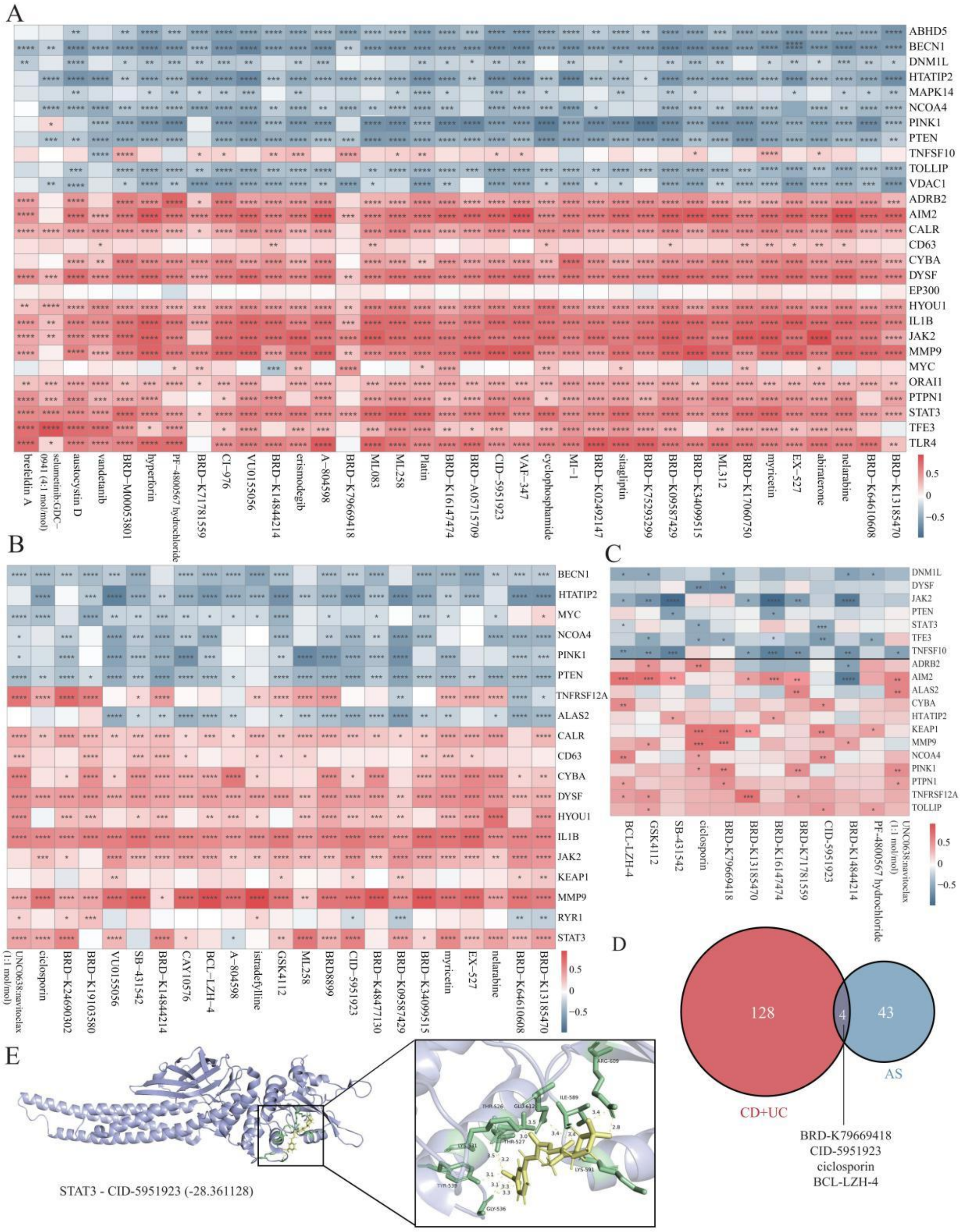

Supplementary Figure S5. Transcriptome-based drug-response prediction and molecular docking of candidate compounds with STAT3. (A–C) Heatmaps showing Pearson correlations between the expression levels of disease-associated Na^+ overload-related genes and the predicted CTRP2 drug-response scores in the (A) CD, (B) UC, and (C) AS datasets. Candidate compounds were prioritized using coverage thresholds of 90% for CD, 80% for UC, and 60% for AS. (D) Overlap of the prioritized compounds across the three disease-specific analyses. Four compounds—ciclosporin, BCL-LZH-4, BRD-K79669418, and CID-5951923—were shared between AS and at least one IBD dataset. (E) Representative predicted binding pose of CID-5951923 within the STAT3 protein structure (PDB ID: 6TLC). The ligand was positioned in a pocket surrounded by THR526, THR527, GLU530, GLY536, VAL537, TYR539, ILE589, SER590, LYS591, GLU612, and ARG609. A potential hydrogen-bond interaction with ARG609 was observed at an interatomic distance of approximately 2.8 Å. The predicted STAT3–CID-5951923 complex yielded a DOCK Grid Score of −28.361128. AS, ankylosing spondylitis; CD, Crohn's disease; CTRP2, Cancer Therapeutics Response Portal version 2; IBD, inflammatory bowel disease; NRG, Na^+ overload-related gene; STAT3, signal transducer and activator of transcription 3; UC, ulcerative colitis.

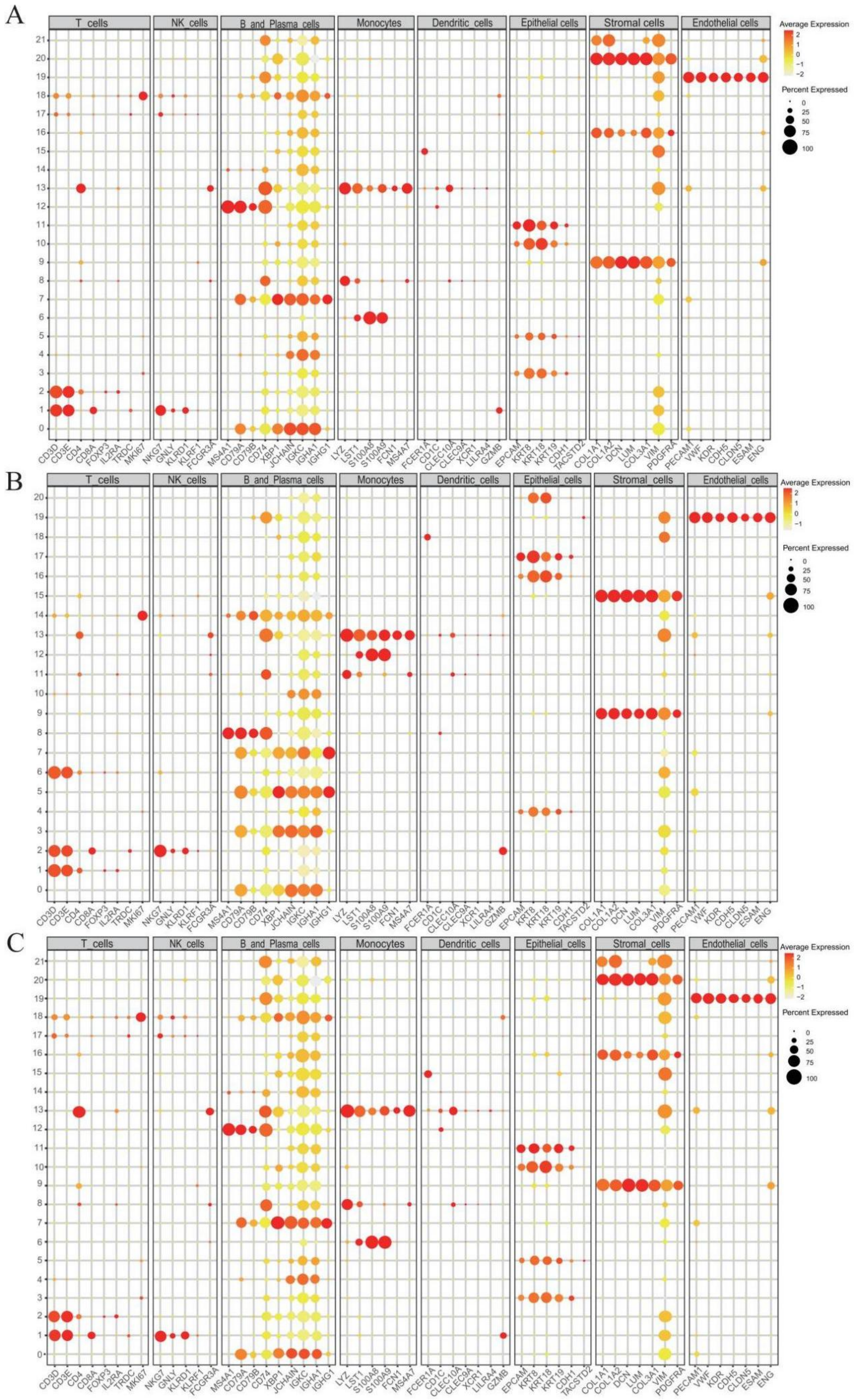
A
B
C
T_cells
NK_cells
B_and_Plasma_cells
Monocytes
Dendritic_cells
Epithelial_cells
Stromal_cells
Endothelial_cells
Average Expression
Percent Expressed
CD3D CD3E CD4 CD8A FOXP3 IL2RA TRDC MKI67
NKG7 GNLY KLRD1 KLRF1 FCGR3A
MS4A1 CD79A CD79B CD74 XBP1 JCHAIN IGKC IGHA1 IGHG1
LYZ LST1 S100A8 S100A9 FCN1 MS4A7
FCER1A CD1C CLEC10A CLEC9A XCR1 LILRA4 GZMB
EPCAM KRT8 KRT18 KRT19 CDH1 TACSTD2
COL1A1 COL1A2 DCN LUM COL3A1 VIM PDGFRA
PECAM1 VWF KDR CDH5 CLDN5 ESAM ENG

Supplementary Figure S6. Bubble plots showing canonical marker-gene expression across annotated cell types in the (A) CD, (B) UC, and (C) AS datasets.

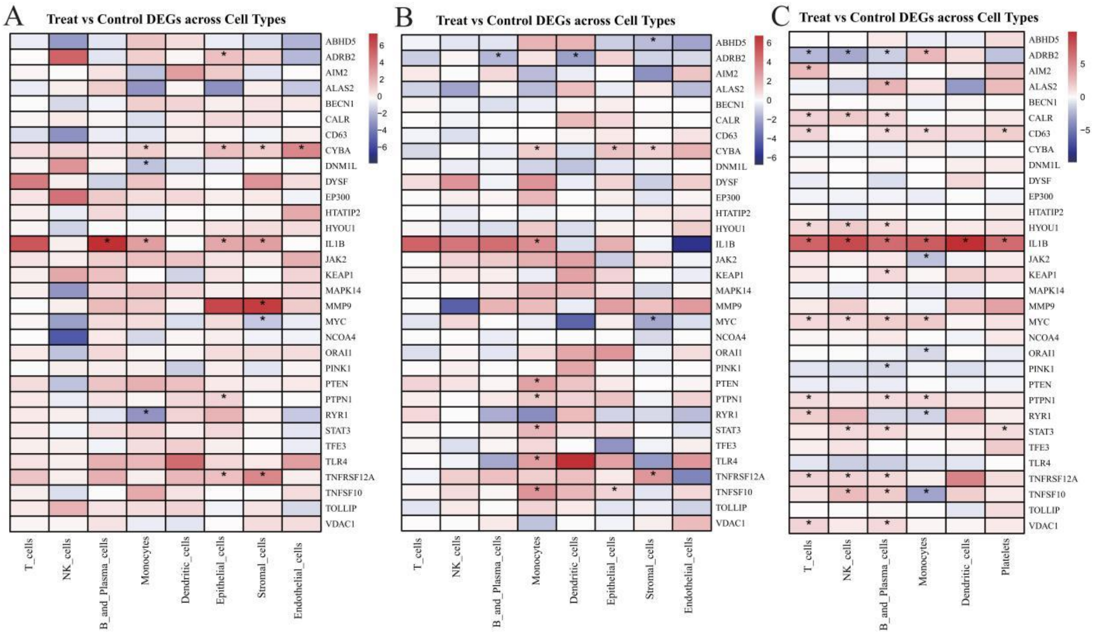

Supplementary Figure S7. Heatmaps showing cell-type-specific differential-expression patterns of the 32 Co-DE-NRGs in the (A) CD, (B) UC, and (C) AS datasets.

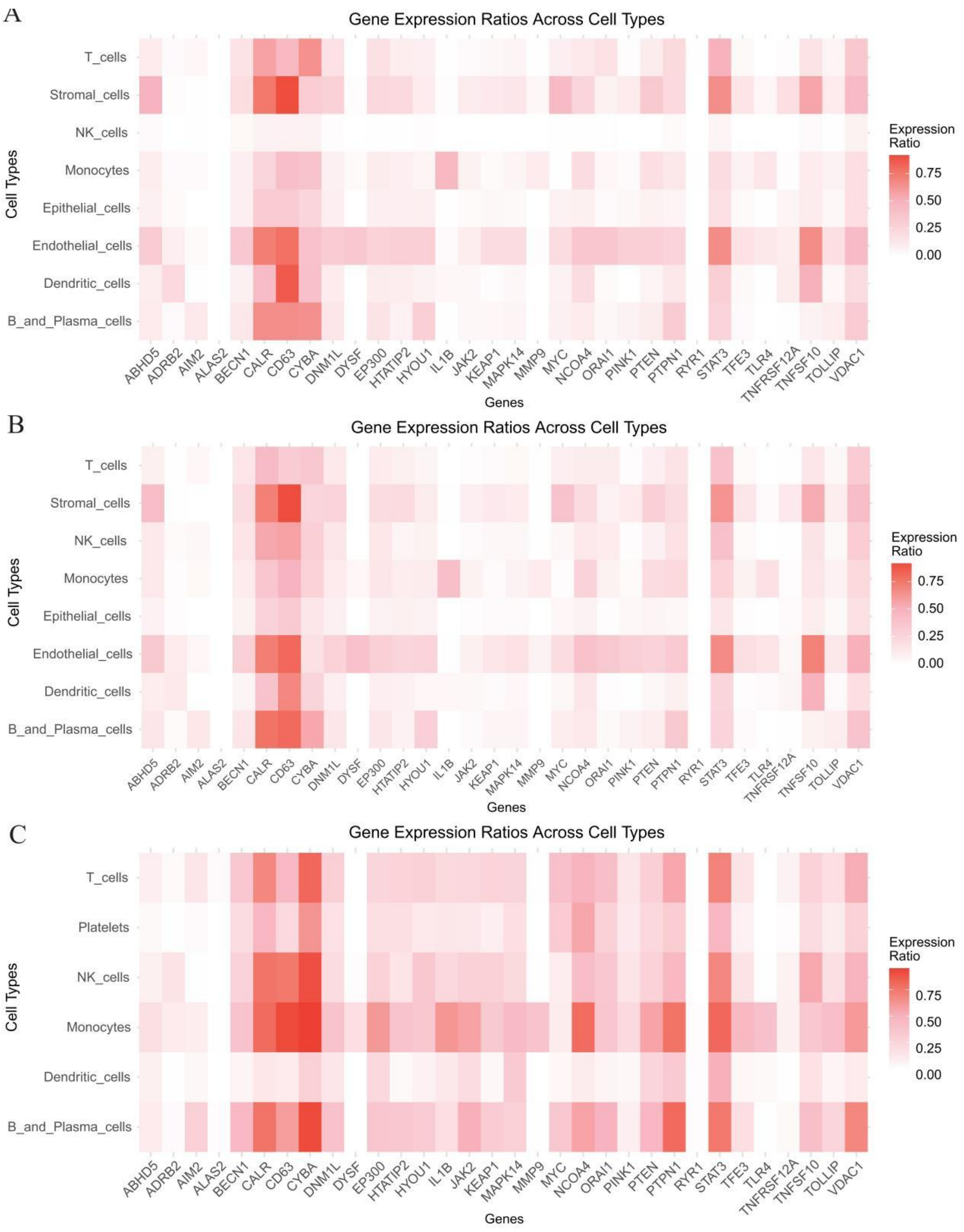

Supplementary Figure S8. Heatmaps showing the expression ratios of the 32 Co-DE-NRGs across annotated cell types in the (A) CD, (B) UC, and (C) AS datasets.

Supplementary Table S1. Complete GeneCards search results for Na^+ overload-induced necrotic death.

Gene Symbol	Description	Category	Uniprot ID	Gifts	GC Id	Relevance score	GeneCards Link
FADD	Fas Associated Via Death Domain	Protein Coding	Q13158	58	GC11P070203	62.2399	https://www.genecards.org/cgi-bin/carddisp.pl?gene=FADD
CASP3	Caspase 3	Protein Coding	P42574	62	GC04M184627	54.3549	https://www.genecards.org/cgi-bin/carddisp.pl?gene=CASP3
CASP8	Caspase 8	Protein Coding	Q14790	64	GC02P201233	53.6321	https://www.genecards.org/cgi-bin/carddisp.pl?gene=CASP8
FAS	Fas Cell Surface Death Receptor	Protein Coding	P25445	63	GC10P134457	48.0526	https://www.genecards.org/cgi-bin/carddisp.pl?gene=FAS
RYR2	Ryanodine Receptor 2	Protein Coding	Q92736	60	GC01P237042	45.3384	https://www.genecards.org/cgi-bin/carddisp.pl?gene=RYR2
TNF	Tumor Necrosis Factor	Protein Coding	P01375	63	GC06P199528	45.0873	https://www.genecards.org/cgi-bin/carddisp.pl?gene=TNF
TP53	Tumor Protein P53	Protein Coding	P04637	66	GC17M007661	43.4957	https://www.genecards.org/cgi-bin/carddisp.pl?gene=TP53
BAX	BCL2 Associated X, Apoptosis Regulator	Protein Coding	Q07812	63	GC19P048954	38.1396	https://www.genecards.org/cgi-bin/carddisp.pl?gene=BAX
SLC9A1	Solute Carrier Family 9 Member A1	Protein Coding	P19634	63	GC01M041363	37.3246	https://www.genecards.org/cgi-bin/carddisp.pl?gene=SLC9A1
FASLG	Fas Ligand	Protein Coding	P48023	60	GC01P172659	37.0844	https://www.genecards.org/cgi-bin/carddisp.pl?gene=FASLG
BAD	BCL2 Associated Agonist Of Cell Death	Protein Coding	Q92934	57	GC11M146126	36.9145	https://www.genecards.org/cgi-bin/carddisp.pl?gene=BAD
CACNA1C	Calcium Voltage-Gated Channel Subunit Alpha1 C	Protein Coding	Q13936	61	GC12P001970	36.1011	https://www.genecards.org/cgi-bin/carddisp.pl?gene=CACNA1C
AIFM1	Apoptosis Inducing Factor	Protein	O95831	63	GC0XM13012	35.6230	https://www.genecards.org/cgi-

Gene Symbol	Description	Category	Uniprot ID	Gifts	GC Id	Relevance score	GeneCards Link
	Mitochondria Associated 1	Coding			9		bin/carddisp.pl?gene=AIFM1
BCL2	BCL2 Apoptosis Regulator	Protein Coding	P10415	64	GC18M063123	35.4694	https://www.genecards.org/cgi-bin/carddisp.pl?gene=BCL2
TNFSF10	TNF Superfamily Member 10	Protein Coding	P50591	58	GC03M172505	34.1042	https://www.genecards.org/cgi-bin/carddisp.pl?gene=TNFSF10
IL6	Interleukin 6	Protein Coding	P05231	63	GC07P022725	34.0666	https://www.genecards.org/cgi-bin/carddisp.pl?gene=IL6
CYCS	Cytochrome C, Somatic	Protein Coding	P99999	59	GC07M025118	33.9760	https://www.genecards.org/cgi-bin/carddisp.pl?gene=CYCS
BCL2L1	BCL2 Like 1	Protein Coding	Q07817	62	GC20M031664	33.7612	https://www.genecards.org/cgi-bin/carddisp.pl?gene=BCL2L1
CASP9	Caspase 9	Protein Coding	P55211	61	GC01M040851	32.2153	https://www.genecards.org/cgi-bin/carddisp.pl?gene=CASP9
PARP1	Poly(ADP-Ribose) Polymerase 1	Protein Coding	P09874	64	GC01M226360	30.7111	https://www.genecards.org/cgi-bin/carddisp.pl?gene=PARP1
AKT1	AKT Serine/Threonine Kinase 1	Protein Coding	P31749	66	GC14M104769	28.7040	https://www.genecards.org/cgi-bin/carddisp.pl?gene=AKT1
SLC8A1	Solute Carrier Family 8 Member A1	Protein Coding	P32418	57	GC02M040078	27.4945	https://www.genecards.org/cgi-bin/carddisp.pl?gene=SLC8A1
ACADM	Acyl-CoA Dehydrogenase Medium Chain	Protein Coding	P11310	58	GC01P075724	27.1015	https://www.genecards.org/cgi-bin/carddisp.pl?gene=ACADM
TLR4	Toll Like Receptor 4	Protein Coding	O00206	63	GC09P117704	26.4407	https://www.genecards.org/cgi-bin/carddisp.pl?gene=TLR4
HIF1A	Hypoxia Inducible Factor 1 Subunit Alpha	Protein Coding	Q16665	62	GC14P061695	26.2684	https://www.genecards.org/cgi-bin/carddisp.pl?gene=HIF1A
MTOR	Mechanistic Target Of Rapamycin	Protein	P42345	68	GC01M011106	26.0791	https://www.genecards.org/cgi-

Gene Symbol	Description	Category	Uniprot ID	Gifts	GC Id	Relevance score	GeneCards Link
	Kinase	Coding					bin/carddisp.pl?gene=MTOR
DNM1L	Dynamin 1 Like	Protein Coding	O00429	60	GC12P032679	25.0470	https://www.genecards.org/cgi-bin/carddisp.pl?gene=DNM1L
SLC5A1	Solute Carrier Family 5 Member 1	Protein Coding	P13866	59	GC22P032043	24.6125	https://www.genecards.org/cgi-bin/carddisp.pl?gene=SLC5A1
APP	Amyloid Beta Precursor Protein	Protein Coding	P05067	63	GC21M025880	24.4272	https://www.genecards.org/cgi-bin/carddisp.pl?gene=APP
CASQ2	Calsequestrin 2	Protein Coding	O14958	57	GC01M115700	24.3592	https://www.genecards.org/cgi-bin/carddisp.pl?gene=CASQ2
IL10	Interleukin 10	Protein Coding	P22301	59	GC01M206767	24.1290	https://www.genecards.org/cgi-bin/carddisp.pl?gene=IL10
IL1B	Interleukin 1 Beta	Protein Coding	P01584	59	GC02M112829	23.9574	https://www.genecards.org/cgi-bin/carddisp.pl?gene=IL1B
TNFRSF1A	TNF Receptor Superfamily Member 1A	Protein Coding	P19438	61	GC12M006328	23.7565	https://www.genecards.org/cgi-bin/carddisp.pl?gene=TNFRSF1A
TGFB1	Transforming Growth Factor Beta 1	Protein Coding	P01137	65	GC19M041301	23.5814	https://www.genecards.org/cgi-bin/carddisp.pl?gene=TGFB1
ACE	Angiotensin I Converting Enzyme	Protein Coding	P12821	64	GC17P063477	23.5750	https://www.genecards.org/cgi-bin/carddisp.pl?gene=ACE
STAT3	Signal Transducer And Activator Of Transcription 3	Protein Coding	P40763	66	GC17M042313	22.6551	https://www.genecards.org/cgi-bin/carddisp.pl?gene=STAT3
VEGFA	Vascular Endothelial Growth Factor A	Protein Coding	P15692	61	GC06P043770	22.6117	https://www.genecards.org/cgi-bin/carddisp.pl?gene=VEGFA
PRKN	Parkin RBR E3 Ubiquitin Protein Ligase	Protein Coding	O60260	61	GC06M161348	21.3475	https://www.genecards.org/cgi-bin/carddisp.pl?gene=PRKN
ALB	Albumin	Protein	P02768	61	GC04P073397	21.0645	https://www.genecards.org/cgi-

Gene Symbol	Description	Category	Uniprot ID	Gifts	GC Id	Relevance score	GeneCards Link
		Coding					bin/carddisp.pl?gene=ALB
HSPA5	Heat Shock Protein Family A (Hsp70) Member 5	Protein Coding	P11021	61	GC09M125234	19.9543	https://www.genecards.org/cgi-bin/carddisp.pl?gene=HSPA5
NFE2L2	NFE2 Like BZIP Transcription Factor 2	Protein Coding	Q16236	64	GC02M177227	19.8452	https://www.genecards.org/cgi-bin/carddisp.pl?gene=NFE2L2
BDNF-AS	BDNF Antisense RNA	RNA Gene		30	GC11P027466	19.7068	https://www.genecards.org/cgi-bin/carddisp.pl?gene=BDNF-AS
JUN	Jun Proto-Oncogene, AP-1 Transcription Factor Subunit	Protein Coding	P05412	63	GC01M058780	19.3795	https://www.genecards.org/cgi-bin/carddisp.pl?gene=JUN
CRP	C-Reactive Protein	Protein Coding	P02741	58	GC01M169920	19.1390	https://www.genecards.org/cgi-bin/carddisp.pl?gene=CRP
CERNA3	Competing Endogenous LncRNA 3 For MiR-645	RNA Gene		21	GC08P056533	19.1252	https://www.genecards.org/cgi-bin/carddisp.pl?gene=CERNA3
NPPA	Natriuretic Peptide A	Protein Coding	P01160	56	GC01M040726	19.0921	https://www.genecards.org/cgi-bin/carddisp.pl?gene=NPPA
NGF	Nerve Growth Factor	Protein Coding	P01138	64	GC01M115285	18.9219	https://www.genecards.org/cgi-bin/carddisp.pl?gene=NGF
PTEN	Phosphatase And Tensin Homolog	Protein Coding	P60484	65	GC10P134441	18.8960	https://www.genecards.org/cgi-bin/carddisp.pl?gene=PTEN
SIRT1	Sirtuin 1	Protein Coding	Q96EB6	62	GC10P067884	18.7468	https://www.genecards.org/cgi-bin/carddisp.pl?gene=SIRT1
BAK1	BCL2 Antagonist/Killer 1	Protein Coding	Q16611	57	GC06M033572	18.5312	https://www.genecards.org/cgi-bin/carddisp.pl?gene=BAK1
GSK3B	Glycogen Synthase Kinase 3 Beta	Protein Coding	P49841	64	GC03M119821	18.4240	https://www.genecards.org/cgi-bin/carddisp.pl?gene=GSK3B
MYC	MYC Proto-Oncogene, BHLH	Protein	P01106	65	GC08P127735	18.4087	https://www.genecards.org/cgi-

Gene Symbol	Description	Category	Uniprot ID	Gifts	GC Id	Relevance score	GeneCards Link
	Transcription Factor	Coding					bin/carddisp.pl?gene=MYC
JAK2	Janus Kinase 2	Protein Coding	O60674	66	GC09P004985	18.3614	https://www.genecards.org/cgi-bin/carddisp.pl?gene=JAK2
HMOX1	Heme Oxygenase 1	Protein Coding	P09601	64	GC22P035380	18.3310	https://www.genecards.org/cgi-bin/carddisp.pl?gene=HMOX1
INS	Insulin	Protein Coding	P01308	60	GC11M002159	18.1320	https://www.genecards.org/cgi-bin/carddisp.pl?gene=INS
NLRP3	NLR Family Pyrin Domain Containing 3	Protein Coding	Q96P20	61	GC01P247741	18.1151	https://www.genecards.org/cgi-bin/carddisp.pl?gene=NLRP3
MAPK14	Mitogen-Activated Protein Kinase 14	Protein Coding	Q16539	64	GC06P199648	17.8428	https://www.genecards.org/cgi-bin/carddisp.pl?gene=MAPK14
PINK1	PTEN Induced Kinase 1	Protein Coding	Q9BXM7	59	GC01P092520	17.5984	https://www.genecards.org/cgi-bin/carddisp.pl?gene=PINK1
SOD2-OT1	SOD2 Overlapping Transcript 1	RNA Gene		19	GC06M159772	17.5173	https://www.genecards.org/cgi-bin/carddisp.pl?gene=SOD2-OT1
SMAD3	SMAD Family Member 3	Protein Coding	P84022	66	GC15P067063	17.0424	https://www.genecards.org/cgi-bin/carddisp.pl?gene=SMAD3
BECN1	Beclin 1	Protein Coding	Q14457	59	GC17M104775	16.8216	https://www.genecards.org/cgi-bin/carddisp.pl?gene=BECN1
RYR1	Ryanodine Receptor 1	Protein Coding	P21817	61	GC19P171589	16.8034	https://www.genecards.org/cgi-bin/carddisp.pl?gene=RYR1
ACTA2	Actin Alpha 2, Smooth Muscle	Protein Coding	P62736	60	GC10M088935	16.2962	https://www.genecards.org/cgi-bin/carddisp.pl?gene=ACTA2
H19	H19 Imprinted Maternally Expressed Transcript	RNA Gene		36	GC11M001995	16.1651	https://www.genecards.org/cgi-bin/carddisp.pl?gene=H19
CTNNB1	Catenin Beta 1	Protein	P35222	66	GC03P041194	16.1631	https://www.genecards.org/cgi-

Gene Symbol	Description	Category	Uniprot ID	Gifts	GC Id	Relevance score	GeneCards Link
		Coding					bin/carddisp.pl?gene=CTNNB1
PRKACA	Protein Kinase CAMP-Activated Catalytic Subunit Alpha	Protein Coding	P17612	65	GC19M115794	16.0708	https://www.genecards.org/cgi-bin/carddisp.pl?gene=PRKACA
MIR21	MicroRNA 21	RNA Gene		33	GC17P059841	16.0679	https://www.genecards.org/cgi-bin/carddisp.pl?gene=MIR21
BNIP3	BCL2 Interacting Protein 3	Protein Coding	Q12983	54	GC10M131966	16.0292	https://www.genecards.org/cgi-bin/carddisp.pl?gene=BNIP3
PTGS2	Prostaglandin-Endoperoxide Synthase 2	Protein Coding	P35354	63	GC01M186671	16.0125	https://www.genecards.org/cgi-bin/carddisp.pl?gene=PTGS2
JPH2	Junctophilin 2	Protein Coding	Q9BR39	55	GC20M044139	15.8334	https://www.genecards.org/cgi-bin/carddisp.pl?gene=JPH2
MFN2	Mitofusin 2	Protein Coding	O95140	60	GC01P011980	15.7998	https://www.genecards.org/cgi-bin/carddisp.pl?gene=MFN2
IGF1	Insulin Like Growth Factor 1	Protein Coding	P05019	60	GC12M102395	15.7955	https://www.genecards.org/cgi-bin/carddisp.pl?gene=IGF1
NOS2	Nitric Oxide Synthase 2	Protein Coding	P35228	61	GC17M027756	15.6933	https://www.genecards.org/cgi-bin/carddisp.pl?gene=NOS2
NOS3	Nitric Oxide Synthase 3	Protein Coding	P29474	62	GC07P170688	15.3475	https://www.genecards.org/cgi-bin/carddisp.pl?gene=NOS3
APOE	Apolipoprotein E	Protein Coding	P02649	62	GC19P171829	14.9173	https://www.genecards.org/cgi-bin/carddisp.pl?gene=APOE
MYLK	Myosin Light Chain Kinase	Protein Coding	Q15746	65	GC03M123610	14.7718	https://www.genecards.org/cgi-bin/carddisp.pl?gene=MYLK
ICAM1	Intercellular Adhesion Molecule 1	Protein Coding	P05362	62	GC19P170923	14.6657	https://www.genecards.org/cgi-bin/carddisp.pl?gene=ICAM1
TARDBP	TAR DNA Binding Protein	Protein	Q13148	59	GC01P091901	14.5053	https://www.genecards.org/cgi-

Gene Symbol	Description	Category	Uniprot ID	Gifts	GC Id	Relevance score	GeneCards Link
		Coding					bin/carddisp.pl?gene=TARDBP
TLR2	Toll Like Receptor 2	Protein Coding	O60603	64	GC04P153684	14.4718	https://www.genecards.org/cgi-bin/carddisp.pl?gene=TLR2
PLA2G6	Phospholipase A2 Group VI	Protein Coding	O60733	60	GC22M092367	14.4581	https://www.genecards.org/cgi-bin/carddisp.pl?gene=PLA2G6
PNPLA2	Patatin Like Domain 2, Triacylglycerol Lipase	Protein Coding	Q96AD5	57	GC11P024751	14.1851	https://www.genecards.org/cgi-bin/carddisp.pl?gene=PNPLA2
PIK3CG	Phosphatidylinositol-4,5-Bisphosphate 3-Kinase Catalytic Subunit Gamma	Protein Coding	P48736	62	GC07P106865	13.9818	https://www.genecards.org/cgi-bin/carddisp.pl?gene=PIK3CG
PPARGC1A	PPARG Coactivator 1 Alpha	Protein Coding	Q9UBK2	60	GC04M023755	13.9299	https://www.genecards.org/cgi-bin/carddisp.pl?gene=PPARGC1A
CCL2	C-C Motif Chemokine Ligand 2	Protein Coding	P13500	60	GC17P034255	13.5401	https://www.genecards.org/cgi-bin/carddisp.pl?gene=CCL2
ENG	Endoglin	Protein Coding	P17813	60	GC09M134668	13.5194	https://www.genecards.org/cgi-bin/carddisp.pl?gene=ENG
TGFBR1	Transforming Growth Factor Beta Receptor 1	Protein Coding	P36897	66	GC09P159927	13.3775	https://www.genecards.org/cgi-bin/carddisp.pl?gene=TGFBR1
FHL1	Four And A Half LIM Domains 1	Protein Coding	Q13642	58	GC0XP136146	13.2626	https://www.genecards.org/cgi-bin/carddisp.pl?gene=FHL1
VDAC1	Voltage Dependent Anion Channel 1	Protein Coding	P21796	59	GC05M133975	13.2481	https://www.genecards.org/cgi-bin/carddisp.pl?gene=VDAC1
SMAD2	SMAD Family Member 2	Protein Coding	Q15796	64	GC18M047809	13.1249	https://www.genecards.org/cgi-bin/carddisp.pl?gene=SMAD2
NPPB	Natriuretic Peptide B	Protein Coding	P16860	55	GC01M040727	13.0968	https://www.genecards.org/cgi-bin/carddisp.pl?gene=NPPB

Gene Symbol	Description	Category	Uniprot ID	Gifts	GC Id	Relevance score	GeneCards Link
ELN	Elastin	Protein Coding	P15502	55	GC07P074027	13.0461	https://www.genecards.org/cgi-bin/carddisp.pl?gene=ELN
MAP1LC3A	Microtubule Associated Protein 1 Light Chain 3 Alpha	Protein Coding	Q9H492	54	GC20P034546	12.9811	https://www.genecards.org/cgi-bin/carddisp.pl?gene=MAP1LC3A
EIF2AK3	Eukaryotic Translation Initiation Factor 2 Alpha Kinase 3	Protein Coding	Q9NZJ5	63	GC02M088556	12.9253	https://www.genecards.org/cgi-bin/carddisp.pl?gene=EIF2AK3
RHOA	Ras Homolog Family Member A	Protein Coding	P61586	62	GC03M049359	12.8615	https://www.genecards.org/cgi-bin/carddisp.pl?gene=RHOA
MAP3K5	Mitogen-Activated Protein Kinase Kinase Kinase 5	Protein Coding	Q99683	62	GC06M136557	12.7865	https://www.genecards.org/cgi-bin/carddisp.pl?gene=MAP3K5
GSDMD	Gasdermin D	Protein Coding	P57764	52	GC08P143553	12.6159	https://www.genecards.org/cgi-bin/carddisp.pl?gene=GSDMD
PGR-AS1	PGR Antisense RNA 1	RNA Gene		19	GC11P131251	12.6034	https://www.genecards.org/cgi-bin/carddisp.pl?gene=PGR-AS1
MMP9	Matrix Metallopeptidase 9	Protein Coding	P14780	66	GC20P046008	12.5891	https://www.genecards.org/cgi-bin/carddisp.pl?gene=MMP9
MIR7-3HG	MIR7-3 Host Gene	RNA Gene	Q8N6C7	33	GC19P170668	12.1766	https://www.genecards.org/cgi-bin/carddisp.pl?gene=MIR7-3HG
TRPM2	Transient Receptor Potential Cation Channel Subfamily M Member 2	Protein Coding	O94759	54	GC21P044350	11.8711	https://www.genecards.org/cgi-bin/carddisp.pl?gene=TRPM2
APOB	Apolipoprotein B	Protein Coding	P04114	58	GC02M020956	11.8575	https://www.genecards.org/cgi-bin/carddisp.pl?gene=APOB
NQO1	NAD(P)H Quinone Dehydrogenase 1	Protein Coding	P15559	61	GC16M069706	11.7979	https://www.genecards.org/cgi-bin/carddisp.pl?gene=NQO1
AGT	Angiotensinogen	Protein Coding	P01019	62	GC01M230690	11.4268	https://www.genecards.org/cgi-bin/carddisp.pl?gene=AGT

Gene Symbol	Description	Category	Uniprot ID	Gifts	GC Id	Relevance score	GeneCards Link
GPT	Glutamic--Pyruvic Transaminase	Protein Coding	P24298	55	GC08P144502	11.4009	https://www.genecards.org/cgi-bin/carddisp.pl?gene=GPT
CYBB	Cytochrome B-245 Beta Chain	Protein Coding	P04839	61	GC0XP037780	11.3964	https://www.genecards.org/cgi-bin/carddisp.pl?gene=CYBB
CDKN3	Cyclin Dependent Kinase Inhibitor 3	Protein Coding	Q16667	54	GC14P066396	11.3694	https://www.genecards.org/cgi-bin/carddisp.pl?gene=CDKN3
ALAS2	5"-Aminolevulinate Synthase 2	Protein Coding	P22557	58	GC0XM055009	11.3009	https://www.genecards.org/cgi-bin/carddisp.pl?gene=ALAS2
SLC7A11	Solute Carrier Family 7 Member 11	Protein Coding	Q9UPY5	58	GC04M138164	11.2996	https://www.genecards.org/cgi-bin/carddisp.pl?gene=SLC7A11
ITPR1	Inositol 1,4,5-Trisphosphate Receptor Type 1	Protein Coding	Q14643	61	GC03P004512	11.1602	https://www.genecards.org/cgi-bin/carddisp.pl?gene=ITPR1
TRPV1	Transient Receptor Potential Cation Channel Subfamily V Member 1	Protein Coding	Q8NER1	61	GC17M003565	10.9885	https://www.genecards.org/cgi-bin/carddisp.pl?gene=TRPV1
LTF	Lactotransferrin	Protein Coding	P02788	58	GC03M046435	10.9604	https://www.genecards.org/cgi-bin/carddisp.pl?gene=LTF
ATG7	Autophagy Related 7	Protein Coding	O95352	57	GC03P030214	10.8571	https://www.genecards.org/cgi-bin/carddisp.pl?gene=ATG7
ACE2	Angiotensin Converting Enzyme 2	Protein Coding	Q9BYF1	61	GC0XM015494	10.8062	https://www.genecards.org/cgi-bin/carddisp.pl?gene=ACE2
CYLD	CYLD Lysine 63 Deubiquitinase	Protein Coding	Q9NQC7	60	GC16P050742	10.7479	https://www.genecards.org/cgi-bin/carddisp.pl?gene=CYLD
PANX1	Pannexin 1	Protein Coding	Q96RD7	58	GC11P094128	10.5370	https://www.genecards.org/cgi-bin/carddisp.pl?gene=PANX1
AIM2	Absent In Melanoma 2	Protein Coding	O14862	53	GC01M169905	10.4823	https://www.genecards.org/cgi-bin/carddisp.pl?gene=AIM2

Gene Symbol	Description	Category	Uniprot ID	Gifts	GC Id	Relevance score	GeneCards Link
NOS1	Nitric Oxide Synthase 1	Protein Coding	P29475	63	GC12M117208	10.4286	https://www.genecards.org/cgi-bin/carddisp.pl?gene=NOS1
PPIF	Peptidylprolyl Isomerase F	Protein Coding	P30405	56	GC10P134338	10.3847	https://www.genecards.org/cgi-bin/carddisp.pl?gene=PPIF
ACTA1	Actin Alpha 1, Skeletal Muscle	Protein Coding	P68133	60	GC01M231082	10.1955	https://www.genecards.org/cgi-bin/carddisp.pl?gene=ACTA1
CXCL10	C-X-C Motif Chemokine Ligand 10	Protein Coding	P02778	57	GC04M076021	10.1399	https://www.genecards.org/cgi-bin/carddisp.pl?gene=CXCL10
ADIPOQ	Adiponectin, C1Q And Collagen Domain Containing	Protein Coding	Q15848	59	GC03P186842	9.9914	https://www.genecards.org/cgi-bin/carddisp.pl?gene=ADIPOQ
CALR	Calreticulin	Protein Coding	P27797	64	GC19P012938	9.8651	https://www.genecards.org/cgi-bin/carddisp.pl?gene=CALR
SLC17A5	Solute Carrier Family 17 Member 5	Protein Coding	Q9NRA2	57	GC06M073593	9.8226	https://www.genecards.org/cgi-bin/carddisp.pl?gene=SLC17A5
HSF1	Heat Shock Transcription Factor 1	Protein Coding	Q00613	59	GC08P144291	9.6724	https://www.genecards.org/cgi-bin/carddisp.pl?gene=HSF1
KCNJ11	Potassium Inwardly Rectifying Channel Subfamily J Member 11	Protein Coding	Q14654	58	GC11M018286	9.6458	https://www.genecards.org/cgi-bin/carddisp.pl?gene=KCNJ11
MIR155	MicroRNA 155	RNA Gene		29	GC21P025573	9.6404	https://www.genecards.org/cgi-bin/carddisp.pl?gene=MIR155
TNFSF12	TNF Superfamily Member 12	Protein Coding	O43508	51	GC17P174536	9.5859	https://www.genecards.org/cgi-bin/carddisp.pl?gene=TNFSF12
PIEZO1	Piezo Type Mechanosensitive Ion Channel Component 1 (Er Blood Group)	Protein Coding	Q92508	51	GC16M088715	9.5813	https://www.genecards.org/cgi-bin/carddisp.pl?gene=PIEZO1
TF	Transferrin	Protein Coding	P02787	64	GC03P145212	9.5168	https://www.genecards.org/cgi-bin/carddisp.pl?gene=TF

Gene Symbol	Description	Category	Uniprot ID	Gifts	GC Id	Relevance score	GeneCards Link
APOA1	Apolipoprotein A1	Protein Coding	P02647	62	GC11M116835	9.4836	https://www.genecards.org/cgi-bin/carddisp.pl?gene=APOA1
MIR221	MicroRNA 221	RNA Gene		29	GC0XM045746	9.4264	https://www.genecards.org/cgi-bin/carddisp.pl?gene=MIR221
ADRB2	Adrenoceptor Beta 2	Protein Coding	P07550	61	GC05P162652	9.4245	https://www.genecards.org/cgi-bin/carddisp.pl?gene=ADRB2
TRAF6	TNF Receptor Associated Factor 6	Protein Coding	Q9Y4K3	58	GC11M036467	9.3262	https://www.genecards.org/cgi-bin/carddisp.pl?gene=TRAF6
PKM	Pyruvate Kinase M1/2	Protein Coding	P14618	63	GC15M072199	9.2608	https://www.genecards.org/cgi-bin/carddisp.pl?gene=PKM
GSR	Glutathione-Disulfide Reductase	Protein Coding	P00390	62	GC08M030678	9.2525	https://www.genecards.org/cgi-bin/carddisp.pl?gene=GSR
HFE	Homeostatic Iron Regulator	Protein Coding	Q30201	57	GC06P026087	9.1890	https://www.genecards.org/cgi-bin/carddisp.pl?gene=HFE
DYSF	Dysferlin	Protein Coding	O75923	55	GC02P071453	9.1449	https://www.genecards.org/cgi-bin/carddisp.pl?gene=DYSF
GPX4	Glutathione Peroxidase 4	Protein Coding	P36969	60	GC19P001103	9.0825	https://www.genecards.org/cgi-bin/carddisp.pl?gene=GPX4
ADRB1	Adrenoceptor Beta 1	Protein Coding	P08588	62	GC10P135157	8.9814	https://www.genecards.org/cgi-bin/carddisp.pl?gene=ADRB1
ATR	ATR Checkpoint Kinase	Protein Coding	Q13535	66	GC03M142449	8.9696	https://www.genecards.org/cgi-bin/carddisp.pl?gene=ATR
MIR222	MicroRNA 222	RNA Gene		29	GC0XM045747	8.9338	https://www.genecards.org/cgi-bin/carddisp.pl?gene=MIR222
TIMP1	TIMP Metallopeptidase Inhibitor 1	Protein Coding	P01033	57	GC0XP062405	8.9222	https://www.genecards.org/cgi-bin/carddisp.pl?gene=TIMP1

Gene Symbol	Description	Category	Uniprot ID	Gifts	GC Id	Relevance score	GeneCards Link
RAB27A	RAB27A, Member RAS Oncogene Family	Protein Coding	P51159	60	GC15M055202	8.8790	https://www.genecards.org/cgi-bin/carddisp.pl?gene=RAB27A
TRPV4	Transient Receptor Potential Cation Channel Subfamily V Member 4	Protein Coding	Q9HBA0	61	GC12M109783	8.8644	https://www.genecards.org/cgi-bin/carddisp.pl?gene=TRPV4
PRKAA1	Protein Kinase AMP-Activated Catalytic Subunit Alpha 1	Protein Coding	Q13131	59	GC05M040759	8.8247	https://www.genecards.org/cgi-bin/carddisp.pl?gene=PRKAA1
FGA	Fibrinogen Alpha Chain	Protein Coding	P02671	62	GC04M154583	8.7469	https://www.genecards.org/cgi-bin/carddisp.pl?gene=FGA
KEAP1	Kelch Like ECH Associated Protein 1	Protein Coding	Q14145	61	GC19M010486	8.7080	https://www.genecards.org/cgi-bin/carddisp.pl?gene=KEAP1
CD36	CD36 Molecule (CD36 Blood Group)	Protein Coding	P16671	63	GC07P080369	8.6661	https://www.genecards.org/cgi-bin/carddisp.pl?gene=CD36
ATP2A2	ATPase Sarcoplasmic/Endoplasmic Reticulum Ca2+ Transporting 2	Protein Coding	P16615	63	GC12P110280	8.6082	https://www.genecards.org/cgi-bin/carddisp.pl?gene=ATP2A2
VIM	Vimentin	Protein Coding	P08670	63	GC10P017227	8.5375	https://www.genecards.org/cgi-bin/carddisp.pl?gene=VIM
CXCR3	C-X-C Motif Chemokine Receptor 3	Protein Coding	P49682	58	GC0XM071615	8.4764	https://www.genecards.org/cgi-bin/carddisp.pl?gene=CXCR3
NLRC4	NLR Family CARD Domain Containing 4	Protein Coding	Q9NPP4	56	GC02M032224	8.4491	https://www.genecards.org/cgi-bin/carddisp.pl?gene=NLRC4
EP300	EP300 Lysine Acetyltransferase	Protein Coding	Q09472	65	GC22P041091	8.4190	https://www.genecards.org/cgi-bin/carddisp.pl?gene=EP300
GNAS	GNAS Complex Locus	Protein Coding	P84996	63	GC20P058839	8.2815	https://www.genecards.org/cgi-bin/carddisp.pl?gene=GNAS
ORAI1	ORAI Calcium Release-Activated Calcium Modulator 1	Protein Coding	Q96D31	57	GC12P141811	8.2721	https://www.genecards.org/cgi-bin/carddisp.pl?gene=ORAI1

Gene Symbol	Description	Category	Uniprot ID	Gifts	GC Id	Relevance score	GeneCards Link
SOST	Sclerostin	Protein Coding	Q9BQB4	55	GC17M043753	8.1153	https://www.genecards.org/cgi-bin/carddisp.pl?gene=SOST
TFEB	Transcription Factor EB	Protein Coding	P19484	55	GC06M115152	8.0132	https://www.genecards.org/cgi-bin/carddisp.pl?gene=TFEB
ABCG2	ATP Binding Cassette Subfamily G Member 2 (JR Blood Group)	Protein Coding	Q9UNQ0	62	GC04M088090	7.8859	https://www.genecards.org/cgi-bin/carddisp.pl?gene=ABCG2
SPP1	Secreted Phosphoprotein 1	Protein Coding	P10451	57	GC04P087975	7.8752	https://www.genecards.org/cgi-bin/carddisp.pl?gene=SPP1
ATF3	Activating Transcription Factor 3	Protein Coding	P18847	57	GC01P212565	7.7800	https://www.genecards.org/cgi-bin/carddisp.pl?gene=ATF3
MMP3	Matrix Metallopeptidase 3	Protein Coding	P08254	62	GC11M102835	7.7688	https://www.genecards.org/cgi-bin/carddisp.pl?gene=MMP3
GATA4	GATA Binding Protein 4	Protein Coding	P43694	61	GC08P011676	7.7387	https://www.genecards.org/cgi-bin/carddisp.pl?gene=GATA4
SIRT3	Sirtuin 3	Protein Coding	Q9NTG7	60	GC11M000215	7.7238	https://www.genecards.org/cgi-bin/carddisp.pl?gene=SIRT3
DPP4	Dipeptidyl Peptidase 4	Protein Coding	P27487	63	GC02M161992	7.6941	https://www.genecards.org/cgi-bin/carddisp.pl?gene=DPP4
TRA-TGC7-1	TRNA-Ala (Anticodon TGC) 7-1	RNA Gene		16	GC06M114794	7.6209	https://www.genecards.org/cgi-bin/carddisp.pl?gene=TRA-TGC7-1
FIS1	Fission, Mitochondrial 1	Protein Coding	Q9Y3D6	51	GC07M101239	7.5882	https://www.genecards.org/cgi-bin/carddisp.pl?gene=FIS1
NCAM1	Neural Cell Adhesion Molecule 1	Protein Coding	P13591	61	GC11P112961	7.5824	https://www.genecards.org/cgi-bin/carddisp.pl?gene=NCAM1
ATF6	Activating Transcription Factor 6	Protein Coding	P18850	61	GC01P161766	7.5517	https://www.genecards.org/cgi-bin/carddisp.pl?gene=ATF6

Gene Symbol	Description	Category	Uniprot ID	Gifts	GC Id	Relevance score	GeneCards Link
SHC1	SHC Adaptor Protein 1	Protein Coding	P29353	57	GC01M154962	7.5071	https://www.genecards.org/cgi-bin/carddisp.pl?gene=SHC1
GH1	Growth Hormone 1	Protein Coding	P01241	56	GC17M063917	7.4963	https://www.genecards.org/cgi-bin/carddisp.pl?gene=GH1
EIF2AK2	Eukaryotic Translation Initiation Factor 2 Alpha Kinase 2	Protein Coding	P19525	62	GC02M037099	7.4741	https://www.genecards.org/cgi-bin/carddisp.pl?gene=EIF2AK2
MIAT	Myocardial Infarction Associated Transcript	RNA Gene		32	GC22P026646	7.3507	https://www.genecards.org/cgi-bin/carddisp.pl?gene=MIAT
DNMT3A	DNA Methyltransferase 3 Alpha	Protein Coding	Q9Y6K1	65	GC02M025228	7.2586	https://www.genecards.org/cgi-bin/carddisp.pl?gene=DNMT3A
JAK1	Janus Kinase 1	Protein Coding	P23458	66	GC01M064833	7.2388	https://www.genecards.org/cgi-bin/carddisp.pl?gene=JAK1
PPARA	Peroxisome Proliferator Activated Receptor Alpha	Protein Coding	Q07869	57	GC22P102866	7.1651	https://www.genecards.org/cgi-bin/carddisp.pl?gene=PPARA
MICU1	Mitochondrial Calcium Uptake 1	Protein Coding	Q9BPX6	55	GC10M072367	7.1408	https://www.genecards.org/cgi-bin/carddisp.pl?gene=MICU1
VIP	Vasoactive Intestinal Peptide	Protein Coding	P01282	57	GC06P201312	7.1364	https://www.genecards.org/cgi-bin/carddisp.pl?gene=VIP
OIP5-AS1	OIP5 Antisense RNA 1	RNA Gene		27	GC15P210389	7.1000	https://www.genecards.org/cgi-bin/carddisp.pl?gene=OIP5-AS1
MCU	Mitochondrial Calcium Uniporter	Protein Coding	Q8NE86	51	GC10P072692	7.0748	https://www.genecards.org/cgi-bin/carddisp.pl?gene=MCU
CYBA	Cytochrome B-245 Alpha Chain	Protein Coding	P13498	58	GC16M088643	6.9925	https://www.genecards.org/cgi-bin/carddisp.pl?gene=CYBA
UMOD	Uromodulin	Protein Coding	P07911	55	GC16M055839	6.8471	https://www.genecards.org/cgi-bin/carddisp.pl?gene=UMOD

Gene Symbol	Description	Category	Uniprot ID	Gifts	GC Id	Relevance score	GeneCards Link
TOLLIP	Toll Interacting Protein	Protein Coding	Q9H0E2	54	GC11M001274	6.7966	https://www.genecards.org/cgi-bin/carddisp.pl?gene=TOLLIP
SLC39A8	Solute Carrier Family 39 Member 8	Protein Coding	Q9C0K1	55	GC04M102252	6.7715	https://www.genecards.org/cgi-bin/carddisp.pl?gene=SLC39A8
CXCL9	C-X-C Motif Chemokine Ligand 9	Protein Coding	Q07325	51	GC04M076001	6.7629	https://www.genecards.org/cgi-bin/carddisp.pl?gene=CXCL9
FKBP1B	FKBP Prolyl Isomerase 1B	Protein Coding	P68106	51	GC02P024033	6.7114	https://www.genecards.org/cgi-bin/carddisp.pl?gene=FKBP1B
ALDH2	Aldehyde Dehydrogenase 2 Family Member	Protein Coding	P05091	63	GC12P111766	6.6339	https://www.genecards.org/cgi-bin/carddisp.pl?gene=ALDH2
TRPC6	Transient Receptor Potential Cation Channel Subfamily C Member 6	Protein Coding	Q9Y210	62	GC11M101451	6.6221	https://www.genecards.org/cgi-bin/carddisp.pl?gene=TRPC6
PRKAB1	Protein Kinase AMP-Activated Non-Catalytic Subunit Beta 1	Protein Coding	Q9Y478	59	GC12P119667	6.5752	https://www.genecards.org/cgi-bin/carddisp.pl?gene=PRKAB1
ITPR3	Inositol 1,4,5-Trisphosphate Receptor Type 3	Protein Coding	Q14573	58	GC06P033620	6.4995	https://www.genecards.org/cgi-bin/carddisp.pl?gene=ITPR3
ISG15	ISG15 Ubiquitin Like Modifier	Protein Coding	P05161	58	GC01P001001	6.4548	https://www.genecards.org/cgi-bin/carddisp.pl?gene=ISG15
ELAVL1	ELAV Like RNA Binding Protein 1	Protein Coding	Q15717	54	GC19M007958	6.4463	https://www.genecards.org/cgi-bin/carddisp.pl?gene=ELAVL1
THBS1	Thrombospondin 1	Protein Coding	P07996	59	GC15P210325	6.3598	https://www.genecards.org/cgi-bin/carddisp.pl?gene=THBS1
MIR494	MicroRNA 494	RNA Gene		26	GC14P123663	6.2713	https://www.genecards.org/cgi-bin/carddisp.pl?gene=MIR494
CTSK	Cathepsin K	Protein Coding	P43235	61	GC01M169599	6.2623	https://www.genecards.org/cgi-bin/carddisp.pl?gene=CTSK

Gene Symbol	Description	Category	Uniprot ID	Gifts	GC Id	Relevance score	GeneCards Link
ABCC2	ATP Binding Cassette Subfamily C Member 2	Protein Coding	Q92887	60	GC10P099782	6.2516	https://www.genecards.org/cgi-bin/carddisp.pl?gene=ABCC2
XBP1	X-Box Binding Protein 1	Protein Coding	P17861	58	GC22M028794	6.1981	https://www.genecards.org/cgi-bin/carddisp.pl?gene=XBP1
MMP13	Matrix Metallopeptidase 13	Protein Coding	P45452	62	GC11M102942	6.1699	https://www.genecards.org/cgi-bin/carddisp.pl?gene=MMP13
RPS6KB1	Ribosomal Protein S6 Kinase B1	Protein Coding	P23443	63	GC17P059893	5.9798	https://www.genecards.org/cgi-bin/carddisp.pl?gene=RPS6KB1
KLF4	KLF Transcription Factor 4	Protein Coding	O43474	60	GC09M107484	5.9721	https://www.genecards.org/cgi-bin/carddisp.pl?gene=KLF4
UCP1	Uncoupling Protein 1	Protein Coding	P25874	57	GC04M140559	5.9506	https://www.genecards.org/cgi-bin/carddisp.pl?gene=UCP1
MIR451A	MicroRNA 451a	RNA Gene		25	GC17M028861	5.8470	https://www.genecards.org/cgi-bin/carddisp.pl?gene=MIR451A
FOXM1	Forkhead Box M1	Protein Coding	Q08050	55	GC12M002857	5.7881	https://www.genecards.org/cgi-bin/carddisp.pl?gene=FOXM1
TGM2	Transglutaminase 2	Protein Coding	P21980	60	GC20M038127	5.7845	https://www.genecards.org/cgi-bin/carddisp.pl?gene=TGM2
ACP1	Acid Phosphatase 1	Protein Coding	P24666	55	GC02P001440	5.7173	https://www.genecards.org/cgi-bin/carddisp.pl?gene=ACP1
HYOU1	Hypoxia Up-Regulated 1	Protein Coding	Q9Y4L1	57	GC11M147049	5.6863	https://www.genecards.org/cgi-bin/carddisp.pl?gene=HYOU1
CX3CL1	C-X3-C Motif Chemokine Ligand 1	Protein Coding	P78423	57	GC16P057372	5.6153	https://www.genecards.org/cgi-bin/carddisp.pl?gene=CX3CL1
TRE-TTC3-1	TRNA-Glu (Anticodon TTC) 3-1	RNA Gene		16	GC01P092347	5.5891	https://www.genecards.org/cgi-bin/carddisp.pl?gene=TRE-TTC3-1

Gene Symbol	Description	Category	Uniprot ID	Gifts	GC Id	Relevance score	GeneCards Link
MIR211	MicroRNA 211	RNA Gene		30	GC15M031065	5.5878	https://www.genecards.org/cgi-bin/carddisp.pl?gene=MIR211
ADM	Adrenomedullin	Protein Coding	P35318	57	GC11P025148	5.5535	https://www.genecards.org/cgi-bin/carddisp.pl?gene=ADM
TNFRSF12A	TNF Receptor Superfamily Member 12A	Protein Coding	Q9NP84	55	GC16P003018	5.5345	https://www.genecards.org/cgi-bin/carddisp.pl?gene=TNFRSF12A
SNORD15A	Small Nucleolar RNA, C/D Box 15A	RNA Gene		25	GC11P075400	5.5196	https://www.genecards.org/cgi-bin/carddisp.pl?gene=SNORD15A
SRF	Serum Response Factor	Protein Coding	P11831	57	GC06P043171	5.4728	https://www.genecards.org/cgi-bin/carddisp.pl?gene=SRF
TRPC3	Transient Receptor Potential Cation Channel Subfamily C Member 3	Protein Coding	Q13507	58	GC04M121879	5.4127	https://www.genecards.org/cgi-bin/carddisp.pl?gene=TRPC3
BRD4	Bromodomain Containing 4	Protein Coding	O60885	60	GC19M115828	5.3999	https://www.genecards.org/cgi-bin/carddisp.pl?gene=BRD4
ATP5F1B	ATP Synthase F1 Subunit Beta	Protein Coding	P06576	57	GC12M060959	5.3409	https://www.genecards.org/cgi-bin/carddisp.pl?gene=ATP5F1B
CX3CR1	C-X3-C Motif Chemokine Receptor 1	Protein Coding	P49238	57	GC03M039279	5.2975	https://www.genecards.org/cgi-bin/carddisp.pl?gene=CX3CR1
FTH1	Ferritin Heavy Chain 1	Protein Coding	P02794	63	GC11M061959	5.2333	https://www.genecards.org/cgi-bin/carddisp.pl?gene=FTH1
PTPN1	Protein Tyrosine Phosphatase Non-Receptor Type 1	Protein Coding	P18031	63	GC20P050510	5.2296	https://www.genecards.org/cgi-bin/carddisp.pl?gene=PTPN1
STAT6	Signal Transducer And Activator Of Transcription 6	Protein Coding	P42226	64	GC12M057095	5.2138	https://www.genecards.org/cgi-bin/carddisp.pl?gene=STAT6
ALPP	Alkaline Phosphatase, Placental	Protein Coding	P05187	57	GC02P232378	5.1821	https://www.genecards.org/cgi-bin/carddisp.pl?gene=ALPP

Gene Symbol	Description	Category	Uniprot ID	Gifts	GC Id	Relevance score	GeneCards Link
CHKA	Choline Kinase Alpha	Protein Coding	P35790	57	GC11M068052	4.9421	https://www.genecards.org/cgi-bin/carddisp.pl?gene=CHKA
HAMP	Hepcidin Antimicrobial Peptide	Protein Coding	P81172	55	GC19P171516	4.9023	https://www.genecards.org/cgi-bin/carddisp.pl?gene=HAMP
MIR185	MicroRNA 185	RNA Gene		31	GC22P102017	4.8529	https://www.genecards.org/cgi-bin/carddisp.pl?gene=MIR185
DVL2	Dishevelled Segment Polarity Protein 2	Protein Coding	O14641	59	GC17M007225	4.6771	https://www.genecards.org/cgi-bin/carddisp.pl?gene=DVL2
SFRP4	Secreted Frizzled Related Protein 4	Protein Coding	Q6FHJ7	55	GC07M037912	4.6004	https://www.genecards.org/cgi-bin/carddisp.pl?gene=SFRP4
SOX2-OT	SOX2 Overlapping Transcript	RNA Gene		31	GC03P180989	4.5138	https://www.genecards.org/cgi-bin/carddisp.pl?gene=SOX2-OT
PWAR4	Prader Willi/Angelman Region RNA 4	RNA Gene		17	GC15P025211	4.4332	https://www.genecards.org/cgi-bin/carddisp.pl?gene=PWAR4
TIMP2	TIMP Metallopeptidase Inhibitor 2	Protein Coding	P16035	54	GC17M078852	4.2745	https://www.genecards.org/cgi-bin/carddisp.pl?gene=TIMP2
CD63	CD63 Molecule	Protein Coding	P08962	57	GC12M060906	4.1900	https://www.genecards.org/cgi-bin/carddisp.pl?gene=CD63
CCR2	C-C Motif Chemokine Receptor 2	Protein Coding	P41597	58	GC03P069069	4.1364	https://www.genecards.org/cgi-bin/carddisp.pl?gene=CCR2
SLC2A4	Solute Carrier Family 2 Member 4	Protein Coding	P14672	60	GC17P174515	4.0833	https://www.genecards.org/cgi-bin/carddisp.pl?gene=SLC2A4
MEF2A	Myocyte Enhancer Factor 2A	Protein Coding	Q02078	58	GC15P099565	3.9663	https://www.genecards.org/cgi-bin/carddisp.pl?gene=MEF2A
EEF1A1	Eukaryotic Translation Elongation Factor 1 Alpha 1	Protein Coding	P68104	57	GC06M115485	3.8958	https://www.genecards.org/cgi-bin/carddisp.pl?gene=EEF1A1

Gene Symbol	Description	Category	Uniprot ID	Gifts	GC Id	Relevance score	GeneCards Link
TFE3	Transcription Factor Binding To IGHM Enhancer 3	Protein Coding	P19532	56	GC0XM049028	3.8827	https://www.genecards.org/cgi-bin/carddisp.pl?gene=TFE3
CTH	Cystathionine Gamma-Lyase	Protein Coding	P32929	61	GC01P096023	3.8690	https://www.genecards.org/cgi-bin/carddisp.pl?gene=CTH
MIR374A	MicroRNA 374a	RNA Gene		26	GC0XM074300	3.8406	https://www.genecards.org/cgi-bin/carddisp.pl?gene=MIR374A
SLN	Sarcolipin	Protein Coding	O00631	46	GC11M146867	3.8312	https://www.genecards.org/cgi-bin/carddisp.pl?gene=SLN
SIRT6	Sirtuin 6	Protein Coding	Q8N6T7	57	GC19M004174	3.7897	https://www.genecards.org/cgi-bin/carddisp.pl?gene=SIRT6
ABHD5	Abhydrolase Domain Containing 5, Lysophosphatidic Acid Acyltransferase	Protein Coding	Q8WTS1	57	GC03P068945	3.6315	https://www.genecards.org/cgi-bin/carddisp.pl?gene=ABHD5
DCN	Decorin	Protein Coding	P07585	60	GC12M091140	3.5835	https://www.genecards.org/cgi-bin/carddisp.pl?gene=DCN
FNDC5	Fibronectin Type III Domain Containing 5	Protein Coding	Q8NAU1	47	GC01M041562	3.5742	https://www.genecards.org/cgi-bin/carddisp.pl?gene=FNDC5
CCR9	C-C Motif Chemokine Receptor 9	Protein Coding	P51686	51	GC03P069054	3.5001	https://www.genecards.org/cgi-bin/carddisp.pl?gene=CCR9
HTATIP2	HIV-1 Tat Interactive Protein 2	Protein Coding	Q9BUP3	51	GC11P025468	3.4286	https://www.genecards.org/cgi-bin/carddisp.pl?gene=HTATIP2
MIR133A1	MicroRNA 133a-1	RNA Gene		26	GC18M040125	3.3914	https://www.genecards.org/cgi-bin/carddisp.pl?gene=MIR133A1
NCOA4	Nuclear Receptor Coactivator 4	Protein Coding	Q13772	51	GC10M046005	3.3740	https://www.genecards.org/cgi-bin/carddisp.pl?gene=NCOA4
MYDGF	Myeloid Derived Growth Factor	Protein Coding	Q969H8	45	GC19M004641	3.3391	https://www.genecards.org/cgi-bin/carddisp.pl?gene=MYDGF

Gene Symbol	Description	Category	Uniprot ID	Gifts	GC Id	Relevance score	GeneCards Link
FBLN2	Fibulin 2	Protein Coding	P98095	55	GC03P030258	3.3123	https://www.genecards.org/cgi-bin/carddisp.pl?gene=FBLN2
MRTFA	Myocardin Related Transcription Factor A	Protein Coding	Q969V6	51	GC22M091582	3.1574	https://www.genecards.org/cgi-bin/carddisp.pl?gene=MRTFA
HSPB6	Heat Shock Protein Family B (Small) Member 6	Protein Coding	O14558	51	GC19M116233	3.1572	https://www.genecards.org/cgi-bin/carddisp.pl?gene=HSPB6
SFRP2	Secreted Frizzled Related Protein 2	Protein Coding	Q96HF1	52	GC04M153780	3.0329	https://www.genecards.org/cgi-bin/carddisp.pl?gene=SFRP2
ID1	Inhibitor Of DNA Binding 1	Protein Coding	P41134	53	GC20P059307	3.0124	https://www.genecards.org/cgi-bin/carddisp.pl?gene=ID1
MTG1	Mitochondrial Ribosome Associated GTPase 1	Protein Coding	Q9BT17	47	GC10P133394	2.9292	https://www.genecards.org/cgi-bin/carddisp.pl?gene=MTG1
KAT2B	Lysine Acetyltransferase 2B	Protein Coding	Q92831	62	GC03P030508	2.8472	https://www.genecards.org/cgi-bin/carddisp.pl?gene=KAT2B
CYGB	Cytoglobin	Protein Coding	Q8WWM9	51	GC17M076527	2.4812	https://www.genecards.org/cgi-bin/carddisp.pl?gene=CYGB
MIR532	MicroRNA 532	RNA Gene		25	GC0XP062582	2.3896	https://www.genecards.org/cgi-bin/carddisp.pl?gene=MIR532
AMFR	Autocrine Motility Factor Receptor	Protein Coding	Q9UKV5	56	GC16M056361	2.2370	https://www.genecards.org/cgi-bin/carddisp.pl?gene=AMFR
HDAC5	Histone Deacetylase 5	Protein Coding	Q9UQL6	61	GC17M044076	2.2281	https://www.genecards.org/cgi-bin/carddisp.pl?gene=HDAC5
SFRP1	Secreted Frizzled Related Protein 1	Protein Coding	Q8N474	55	GC08M041262	1.9306	https://www.genecards.org/cgi-bin/carddisp.pl?gene=SFRP1
NOX5	NADPH Oxidase 5	Protein Coding	Q96PH1	50	GC15P210903	1.7279	https://www.genecards.org/cgi-bin/carddisp.pl?gene=NOX5

Gene Symbol	Description	Category	Uniprot ID	Gifts	GC Id	Relevance score	GeneCards Link
AOX1	Aldehyde Oxidase 1	Protein Coding	Q06278	54	GC02P200585	1.6967	https://www.genecards.org/cgi-bin/carddisp.pl?gene=AOX1
MYL7	Myosin Light Chain 7	Protein Coding	Q01449	50	GC07M044138	1.2011	https://www.genecards.org/cgi-bin/carddisp.pl?gene=MYL7
SDC4	Syndecan 4	Protein Coding	P31431	54	GC20M045325	1.0790	https://www.genecards.org/cgi-bin/carddisp.pl?gene=SDC4
LOC129391106	MPRA-Validated Peak3472 Silencer	Functional Element		8	GC19P176351	1.0068	https://www.genecards.org/cgi-bin/carddisp.pl?gene=LOC129391106

Note. GeneCards was searched on 23 April 2026 using the exact keyword "Na^+ overload-induced necrotic death." The complete export contains 263 genes, ordered by decreasing relevance score. Full URLs are shown to support direct clicking or copy-and-paste when a document viewer does not activate embedded hyperlinks.

Supplementary Table S2. Nominal p-values and FDR-adjusted q-values for differential expression between normal and diseased samples in CD, UC, and AS whole blood.

	CD		UC		AS-Blood			CD		UC		AS-Blood	
**Gene**	**pvalue**	**FDR**	**pvalue**	**FDR**	**pvalue**	**FDR**	**Gene**	**pvalue**	**FDR**	**pvalue**	**FDR**	**pvalue**	**FDR**
**ABCC2**	9.7e-05	0.0012	0.9215	0.9632	0.2818	0.5008	**ISG15**	0.0002	0.0025	1e-06	9e-06	0.1724	0.3623
**ABHD5**	0.4893	0.6713	0.0004	0.0016	0.0034	0.0400	**ITPR1**	0.9189	0.9416	NA	NA	0.0837	0.2482
**ACAD**	0.0038	0.0180	NA	NA	0.3821	0.6280	**ITPR3**	0.2359	0.3887	0.4430	0.5701	0.0101	0.0624
**ACP1**	0.0747	0.1907	0.0073	0.0197	0.4117	0.6508	**JAK1**	0.0418	0.1220	NA	NA	0.6976	0.8331
**ACTA2**	0.0264	0.0843	NA	NA	0.5640	0.7620	**JAK2**	0.0087	0.0355	1e-06	7e-06	0.0024	0.0400

**ADIPO**	4.5e-05	0.0008	0.9542	0.9750	0.5497	0.7605	**JPH2**	0.5243	0.6963	0.9476	0.9750	0.5265	0.7345
**ADM**	0.0017	0.0104	NA	NA	0.0959	0.2654	**JUN**	0.0409	0.1215	0.3654	0.4951	0.7697	0.8799
**ADRB2**	0.7301	0.8245	8.5e-05	0.0004	3.5e-05	0.0014	**KEAP1**	0.0060	0.0266	0.6907	0.7930	0.0479	0.1623
**AIFM1**	0.4798	0.6664	8e-06	4.6e-05	0.0959	0.2654	**KLF4**	0.4285	0.6029	0.0994	0.1897	0.1531	0.3344
**AIM2**	0.1275	0.2644	1.2e-05	6.5e-05	0.0141	0.0712	**LTF**	0.0732	0.1899	0.0003	0.0016	0.6003	0.7785
**AKT1**	0.1200	0.2523	0.4198	0.5445	0.9536	0.9593	**MAP1LC**	0.0097	0.0376	1.2e-05	6.4e-05	0.2285	0.4463
**ALAS2**	0.0002	0.0025	0.9651	0.9768	0.0050	0.0469	**MAP3K5**	0.6128	0.7536	NA	NA	0.1747	0.3627
**ALDH2**	0.9915	0.9915	1e-04	0.0004	0.1523	0.3344	**MAPK14**	0.6984	0.8021	0.0229	0.0553	0.0319	0.1206
**ALPP**	0.0090	0.0355	0.0343	0.0757	0.8606	0.9225	**MEF2A**	0.1467	0.2832	0.6796	0.7890	0.9031	0.9370
**AMFR**	0.8374	0.9027	0.8718	0.9337	0.7954	0.8862	**MFN2**	0.1510	0.2881	0.0090	0.0239	0.1095	0.2693
**APOA1**	3e-05	0.0006	0.1449	0.2540	0.5712	0.7620	**MMP9**	0.0013	0.0094	1e-06	9e-06	0.0211	0.0925
**APP**	0.1193	0.2523	0.1453	0.2540	0.0059	0.0469	**MTG1**	0.5223	0.6963	0.0667	0.1347	0.4813	0.7134
**ATF3**	0.0007	0.0070	0.0032	0.0097	0.5930	0.7751	**MYC**	0.0270	0.0847	0.0346	0.0757	0.0121	0.0698
**ATF6**	0.0835	0.1978	0.3283	0.4656	0.0289	0.1166	**MYLK**	0.2167	0.3708	0.7887	0.8786	0.1648	0.3508
**ATG7**	0.8660	0.9066	0.7521	0.8493	0.0491	0.1632	**NCAM1**	0.7006	0.8021	0.7283	0.8281	0.0695	0.2139
**ATP2A**	0.0986	0.2182	0.0070	0.0192	0.1492	0.3344	**NCOA4**	7.2e-05	0.0009	NA	NA	0.0005	0.0136
**ATR**	0.6894	0.8021	0.3845	0.5106	0.1003	0.2660	**NFE2L2**	0.0703	0.1853	0.0123	0.0319	0.0799	0.2413
**BAD**	0.0127	0.0481	0.0554	0.1161	0.0645	0.2022	**NLRC4**	0.3485	0.5165	0.2104	0.3391	0.0188	0.0869
**BAK1**	3e-06	0.0001	0.0243	0.0561	0.3706	0.6278	**NLRP3**	0.5854	0.7513	0.1459	0.2540	0.1071	0.2693
**BAX**	0.0178	0.0604	0.2353	0.3651	0.6837	0.8331	**NOS3**	0.8684	0.9066	0.0021	0.0071	0.4403	0.6706

**BCL2**	0.8446	0.9045	0.8311	0.9076	0.2800	0.5008	**NQO1**	0.7693	0.8571	0.5995	0.7245	0.9872	0.9872
**BCL2L**	0.1626	0.3032	0.6696	0.7852	0.9199	0.9485	**ORAI1**	0.0803	0.1978	0.0026	0.0083	0.0092	0.0624
**BECN1**	1.5e-05	0.0003	0.0004	0.0016	0.0076	0.0553	**PANX1**	0.6738	0.7989	0.5612	0.6900	0.9536	0.9593
**BNIP3**	3e-06	0.0001	0.1468	0.2540	0.3821	0.6280	**PARP1**	0.0813	0.1978	0.6716	0.7852	0.0038	0.0400
**BRD4**	0.0694	0.1853	0.5456	0.6759	0.5149	0.7244	**PIK3CG**	0.1644	0.3033	0.3668	0.4951	0.2896	0.5008
**CALR**	0.0017	0.0104	NA	NA	0.0399	0.1443	**PINK1**	0.0004	0.0043	NA	NA	0.0058	0.0469
**CASP3**	0.6583	0.7862	0.1550	0.2653	0.2411	0.4654	**PLA2G6**	0.8161	0.8880	0.3283	0.4656	0.4056	0.6475
**CASP8**	0.6043	0.7513	0.3475	0.4848	0.4056	0.6475	**PNPLA2**	0.6561	0.7862	0.0058	0.0165	0.6131	0.7795
**CASP9**	0.8541	0.9066	0.6081	0.7245	0.0139	0.0712	**PPARA**	0.0614	0.1671	1e-05	5.5e-05	0.4530	0.6809
**CCL2**	0.4197	0.6007	7.1e-05	0.0003	0.7792	0.8799	**PPIF**	0.6805	0.8011	0.8524	0.9189	0.1115	0.2693
**CCR2**	0.0772	0.1942	0.0239	0.0560	0.7072	0.8385	**PRKAA1**	0.2993	0.4601	0.1243	0.2319	0.1245	0.2912
**CCR9**	6.9e-05	0.0009	0.4728	0.5947	0.0856	0.2494	**PRKAB1**	0.8636	0.9066	5e-06	3e-05	0.4924	0.7233
**CD36**	0.0138	0.0490	0.3310	0.4656	0.2587	0.4877	**PRKACA**	0.4250	0.6029	0.4695	0.5947	0.4034	0.6475
**CD63**	0.0010	0.0085	8e-06	4.6e-05	0.0311	0.1202	**PTEN**	1.5e-05	0.0003	NA	2e-06	NA	5.7e-05
**CHKA**	0.0009	0.0083	NA	NA	0.8283	0.9105	**PTGS2**	0.0040	0.0180	0.4653	0.5942	0.1025	0.2660
**CTH**	0.0027	0.0146	NA	2e-06	0.7214	0.8433	**PTPN1**	0.2719	0.4341	0.0302	0.0687	0.0135	0.0712
**CTNNB**	0.4007	0.5835	0.2418	0.3683	0.5081	0.7244	**RAB27A**	0.5184	0.6963	0.2413	0.3683	0.0067	0.0509
**CTSK**	0.1662	0.3033	0.0001	0.0004	0.8780	0.9225	**RHOA**	0.7646	0.8571	0.1390	0.2509	0.4991	0.7237
**CX3CR**	0.3361	0.5119	0.4813	0.6008	2.3e-05	0.0012	**RPS6KB1**	0.0072	0.0308	1e-04	0.0004	0.5763	0.7620
**CXCL1**	0.0013	0.0094	5.8e-05	0.0002	0.6301	0.7924	**RYR1**	0.0033	0.0169	0.9542	0.9750	0.0178	0.0848

**CXCR3**	0.5854	0.7513	0.1834	0.3014	0.0002	0.0095	**SDC4**	0.4817	0.6664	0.6927	0.7930	0.2615	0.4877
**CYBA**	0.0015	0.0099	4.1e-05	0.0002	0.0098	0.0624	**SFRP1**	0.8756	0.9084	0.8460	0.9179	0.6151	0.7795
**CYBB**	0.1846	0.3261	0.2190	0.3462	0.1887	0.3752	**SFRP2**	1.4e-05	0.0003	0.0559	0.1161	0.6915	0.8331
**CYCS**	6.8e-05	0.0009	NA	NA	0.1553	0.3349	**SHC1**	0.0534	0.1489	0.1772	0.2971	0.0119	0.0698
**CYLD**	0.8185	0.8880	0.0006	0.0023	0.4553	0.6809	**SIRT1**	0.6064	0.7513	0.2185	0.3462	0.4177	0.6542
**DNM1L**	0.1719	0.3069	0.0134	0.0339	0.0394	0.1443	**SIRT3**	0.2951	0.4601	0.0030	0.0094	0.8365	0.9136
**DNMT3**	0.8948	0.9226	0.9118	0.9632	0.1119	0.2693	**SIRT6**	0.2282	0.3827	1e-06	1e-05	0.8036	0.8893
**DPP4**	0.0885	0.2012	0.9226	0.9632	0.0936	0.2654	**SLC17A5**	0.2450	0.3988	0.0186	0.0463	0.2866	0.5008
**DVL2**	0.3595	0.5281	0.1669	0.2827	0.0504	0.1641	**SLC39A8**	0.1442	0.2816	0.6062	0.7245	0.8864	0.9254
**DYSF**	0.0229	0.0746	0.0131	0.0335	0.0172	0.0842	**SLC9A1**	0.4162	0.6007	0.2698	0.4000	0.8689	0.9225
**EEF1A**	0.0216	0.0719	0.1270	0.2343	0.8689	0.9225	**SMAD2**	0.0925	0.2076	0.9847	0.9847	0.2587	0.4877
**EIF2A**	0.2979	0.4601	0.0947	0.1829	0.3799	0.6280	**SMAD3**	0.5164	0.6963	0.3058	0.4414	0.0003	0.0108
**EIF2A**	0.7006	0.8021	0.7939	0.8786	0.1092	0.2693	**SPP1**	NA	2e-05	0.0335	0.0751	0.4239	0.6576
**ELAVL**	0.1062	0.2321	0.3981	0.5204	0.0200	0.0899	**SRF**	0.9406	0.9463	0.0573	0.1175	0.6605	0.8183
**ENG**	0.2937	0.4601	0.0817	0.1597	0.0037	0.0400	**STAT3**	NA	5e-06	1e-06	6e-06	0.0410	0.1449
**EP300**	0.2224	0.3767	0.0426	0.0918	0.0099	0.0624	**STAT6**	0.0037	0.0180	0.2451	0.3700	0.8689	0.9225
**FADD**	0.1376	0.2787	0.0059	0.0165	0.1782	0.3652	**TARDBP**	0.2122	0.3669	0.1825	0.3014	0.0258	0.1073
**FAS**	0.9309	0.9463	0.5986	0.7245	0.0059	0.0469	**TFE3**	0.0862	0.1988	0.0197	0.0482	0.0037	0.0400
**FASLG**	0.5443	0.7115	0.3749	0.5019	0.6452	0.8053	**TFEB**	0.5980	0.7513	0.6110	0.7245	0.1299	0.2995
**FGA**	0.1607	0.3032	0.3973	0.5204	0.8780	0.9225	**TGFB1**	0.3422	0.5165	0.9847	0.9847	0.9283	0.9512

**FHL1**	0.7973	0.8765	0.2025	0.3296	0.5149	0.7244	**TGM2**	0.5938	0.7513	0.0030	0.0094	0.5013	0.7237
**FIS1**	0.196	0.3424	0.1087	0.2051	0.6682	0.8216	**THBS1**	0.3485	0.5165	0.8869	0.9437	0.1898	0.3752
**FKBP1**	0.0028	0.0146	0.0033	0.0098	0.5784	0.7620	**TIMP1**	0.0846	0.1978	1e-06	9e-06	0.3572	0.6113
**FTH1**	0.0453	0.1298	0.0009	0.0031	0.1851	0.3748	**TIMP2**	0.9406	0.9463	0.1312	0.2393	0.4403	0.6706
**GNAS**	0.6192	0.7559	0.2687	0.4000	0.6976	0.8331	**TLR2**	0.7856	0.8694	0.0023	0.0075	0.2896	0.5008
**GPX4**	0.1425	0.2816	0.0005	0.0021	0.2800	0.5008	**TLR4**	0.0350	0.1058	0.0001	0.0007	0.0012	0.0222
**GSK3B**	0.2488	0.4009	0.8077	0.8879	0.0137	0.0712	**TNF**	0.0538	0.1489	1e-06	9e-06	0.0557	0.1779
**GSR**	0.0012	0.0094	0.0236	0.0560	0.2866	0.5008	**TNFRSF1**	0.0039	0.0180	0.3668	0.4951	0.0295	0.1166
**HDAC5**	0.6561	0.7862	0.0050	0.0143	0.7954	0.8862	**TNFRSF1**	0.0021	0.0118	0.0454	0.0967	0.5690	0.7620
**HIF1A**	0.0089	0.0355	NA	NA	0.0979	0.2660	**TNFSF10**	0.1116	0.2406	6e-04	0.0022	0.0036	0.0400
**HMOX**	0.5383	0.7092	0.0789	0.1560	0.7389	0.8577	**TOLLIP**	0.6064	0.7513	0.0016	0.0054	0.0471	0.1623
**HSF1**	0.2365	0.3887	0.2819	0.4141	0.1376	0.3129	**TP53**	0.7233	0.8224	0.3058	0.4414	0.0010	0.0221
**HSPA5**	0.0835	0.1978	0.0007	0.0028	0.7792	0.8799	**TRAF6**	0.0174	0.0602	0.0001	0.0005	0.7469	0.8610
**HTATI**	0.0135	0.0488	0.0034	0.0100	6e-06	0.0005	**TRPM2**	0.1700	0.3068	0.0673	0.1347	0.5640	0.7620
**HYOU1**	0.0009	0.0084	NA	NA	0.0034	0.0400	**VDAC1**	0.0335	0.1030	4e-06	2.7e-05	0.0055	0.0469
**IL10**	0.0018	0.0109	0.2342	0.3651	0.7214	0.8433	**VEGFA**	0.1434	0.2816	0.3518	0.4866	0.1025	0.2660
**IL1B**	0.0021	0.0118	NA	1e-06	0.0224	0.0955	**VIM**	0.0011	0.0091	0.9574	0.9750	0.6057	0.7794
**INS**	0.1290	0.2644	0.7719	0.8658	0.9536	0.9593	**XBP1**	0.0133	0.0488	NA	NA	0.1240	0.2912

Supplementary Table S3. Performance of representative machine-learning models in model-development and held-out GEO cohorts.

Disease	Representative model	GEO accession(s)	Control, n	Disease, n	Total, n
CD	XGBoost	GSE16879; GSE36807; GSE59071	24	58	82
CD	XGBoost	GSE179285	23	86	109
UC	Random forest	GSE13367; GSE59071; GSE75214	42	228	270
UC	Random forest	GSE179285	23	55	78
AS	Stepglm[backward]+RF	GSE18781	25	18	43
AS	Stepglm[backward]+RF	GSE25101	16	16	32

AUC (95% CI)	Accuracy	Sensitivity	Specificity	Precision	F1 score
0.994 (0.980-1.000)	0.927	0.897	1.000	1.000	0.945
0.813 (0.708-0.901)	0.706	0.698	0.739	0.909	0.789
0.951 (0.924-0.973)	0.841	0.811	1.000	1.000	0.896
0.832 (0.733-0.915)	0.718	0.709	0.739	0.867	0.780
0.956 (0.891-1.000)	0.907	0.944	0.880	0.850	0.895
0.773 (0.594-0.926)	0.688	0.813	0.563	0.650	0.722

Supplementary Table S4. Candidate compounds prioritized by transcriptome-based drug-response prediction and exploratory molecular docking against STAT3.

Candidate compound	Prioritized dataset(s)	STAT3 docking status	DOCK Grid Score	Predicted interaction
Ciclosporin	UC and AS	Not evaluated	NA	NA
BCL-LZH-4	UC and AS	Completed	−35.321896	No conventional hydrogen bond was identified under the applied visualization criteria
BRD-K79669418	CD and AS	Incomplete	NA	No completed ligand-growth pose was generated
CID-5951923	CD, UC, and AS	Completed and visualized	−28.361128	Potential hydrogen bond with ARG609 at approximately 2.8 Å

Abbreviations: AS, ankylosing spondylitis; CD, Crohn's disease; IBD, inflammatory bowel disease; NA, not applicable or not available; STAT3, signal transducer and activator of transcription 3; UC, ulcerative colitis. Candidate compounds were prioritized using disease-specific coverage criteria based on the correlations between NRG expression and predicted CTRP2 drug-response scores. Docking results represent computationally predicted binding poses and should not be interpreted as evidence of biochemical binding or therapeutic efficacy.
